# LpCbDR1 regulates leaf senescence and drought tolerance by activating the chlorophyll b reductase gene and stress-related genes in perennial ryegrass

**DOI:** 10.1093/hr/uhag093

**Published:** 2026-03-09

**Authors:** Huanhuan Hao, Qi Zhou, Tingchao Yin, Chenxu Dong, Ziyi Zhang, Yingjun Chi, Jing Zhang, Bin Xu

**Affiliations:** College of Agro-grassland Science, Nanjing Agricultural University, Nanjing, Jiangsu 210095, China; College of Agro-grassland Science, Nanjing Agricultural University, Nanjing, Jiangsu 210095, China; College of Agro-grassland Science, Nanjing Agricultural University, Nanjing, Jiangsu 210095, China; College of Agro-grassland Science, Nanjing Agricultural University, Nanjing, Jiangsu 210095, China; College of Agro-grassland Science, Nanjing Agricultural University, Nanjing, Jiangsu 210095, China; College of Agro-grassland Science, Nanjing Agricultural University, Nanjing, Jiangsu 210095, China; College of Agro-grassland Science, Nanjing Agricultural University, Nanjing, Jiangsu 210095, China; College of Agro-grassland Science, Nanjing Agricultural University, Nanjing, Jiangsu 210095, China

## Abstract

Leaf chlorosis and senescence are key indicators of prolonged drought stress. In this study, we found that suppressing the chlorophyll *b* reductase gene (*LpNOL*) delayed drought-induced leaf chlorosis in perennial ryegrass (*Lolium perenne*). Through a yeast one-hybrid (Y1H) library screen, we identified a NAC transcription factor, designated chlorophyll b degradation regulator 1 (LpCbDR1), as a direct activator of *LpNOL*. Subcellular localization analysis confirmed that LpCbDR1 localizes to the nucleus, and its direct binding to the *LpNOL* promoter was validated by electrophoretic mobility shift assay (EMSA) and CUT&Tag-qPCR assays. Overexpression of *LpCbDR1* accelerated leaf senescence, whereas knockdown of *LpCbDR1* delayed leaf senescence. Notably, *LpCbDR1*’s expression was not only upregulated during leaf senescence but also induced by osmotic stress, promoting further investigation into its role and underlying mechanisms in regulating drought tolerance. Phenotypic analysis showed that *LpCbDR1*-overexpressing lines exhibited significantly higher drought tolerance compared to wild-type (WT) plants, while *LpCbDR1*-*RNAi* lines were drought-sensitive than WT. Integrated RNA-seq and CUT&Tag analysis identified *LpPLA7* and *LpERF1B* as downstream targets of LpCbDR1. Directly binding of LpCbDR1 to the promoter of *LpPLA7* and *LpERF1B* was confirmed by Y1H, EMSA, and CUT&Tag-qPCR assays. Both *LpPLA7* and *LpERF1B* were drought-inducible, and functional validation revealed that overexpression of either gene enhanced osmotic stress tolerance in both WT and *LpCbDR1*-*RNAi* backgrounds. Collectively, this study demonstrates that LpCbDR1 regulates natural, dark-, and drought-induced leaf senescence by activating *LpNOL*, and improves drought tolerance at least partially through direct activation of *LpPLA7* and *LpERF1B* in perennial ryegrass.

## Introduction

Perennial ryegrass (*Lolium perenne* L.) is a popular perennial grass grown globally for turf and forage purposes [1]. Maintaining its green canopy is crucial for turf quality in amenity and sports settings. Perennial ryegrass is sensitive to drought, which can lead to leaf yellowing, premature leaf senescence, and degradation of turf/lawn quality. Studying the molecular mechanisms of drought-induced leaf senescence and drought tolerance is essential for enhancing the genetic traits of perennial ryegrass and other turf grass species.

Leaf chlorosis and senescence are important signs of prolonged drought stress, causing a decrease in green leaves in perennial ryegrass [2]. Accelerated chlorophyll (Chl) degradation causes Chl loss in stress-induced leaves [3]. The degradation of Chl *b* is the initial step in the Chl catabolic pathway (also known as the pheophorbide *a* oxygenase [PAO]/phyllobilin pathway), which is catabolized by two isoenzymes non-yellowing color 1 (NYC1) and NYC1-like (NOL) [4, 5]. Expression of *NOL* was high in leaves at late stages of senescence [6, 7] and induced by abiotic stresses in various plant species [7, 8]. Understanding the regulatory cascades of *NOL* might provide more insights into the regulation of age-dependent and stress-induced leaf senescence.

During drought, senescence helps plants conserve water and transfer nutrients, but excessive senescence can hinder growth and development [9]. Leaf senescence is finely regulated and is often associated with enhanced drought tolerance. For example, overexpression of a cytokinin synthesis gene (*IPT*) under a senescence-specific promoter delayed leaf senescence and improved drought tolerance in various plant species [10–12]. The Arabidopsis NAC transcription factor NTL4 (NAC with transmembrane motif 1-like 4) was highly responsive to drought stress, with null mutants showing delayed drought-induced leaf senescence and improved drought tolerance [13]. Similarly, another transcription factor, JUNGBRUNNEN1 (ANAC042), inhibits leaf senescence and enhances drought tolerance in plants [14, 15]. Conversely, certain transcription factors, such as RD26 (RESPONSIVE TO DESICCATION 26), can transactivate stress- and senescence-associated genes to promote both senescence and drought tolerance [16–22]. These findings suggest a complex relationship between leaf senescence and drought stress, with potential for improving drought tolerance through manipulation of senescence-related genes.

Despite the importance of precise regulation of leaf senescence during drought stress, the impact of Chl degradation on plant drought tolerance is not fully understood [23]. This study aims to investigate the role of the Chl *b* reductase gene (*LpNOL*) in drought responses and to identify the upstream transcription factors regulating *LpNOL* in perennial ryegrass. This research will provide insights into how the transcription factor-*LpNOL* module coordinates leaf senescence and drought tolerance in perennial ryegrass and other plant species.

## Results

### Senescence-inducible LpCbDR1 directly targets *LpNOL* and transactivates its expression

Our previous study has shown that knockdown of *LpNOL* delays dark- and heat-induced leaf senescence in perennial ryegrass [7]. To investigate the impact of inhibiting Chl *b* degradation on drought response, we subjected *LpNOL-RNAi* transgenic lines to drought stress. As shown in Fig. S1, following drought treatment, leaves of *LpNOL-RNAi* lines remained green, with significantly higher total Chl content and Chl *b*/Chl *a* ratio compared to WT plants (Fig. S1). These results indicate that suppressing *LpNOL* delays drought-induced leaf chlorosis in perennial ryegrass.

**Figure 1:**
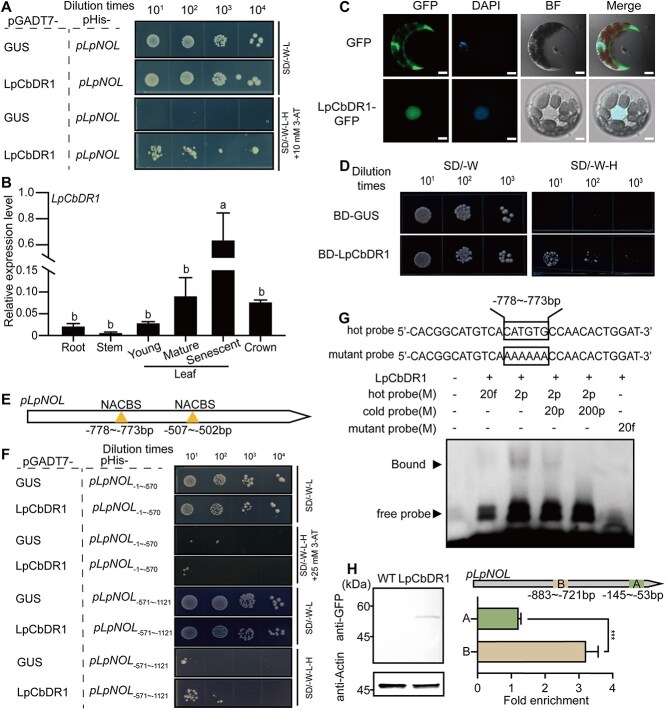
LpCbDR1 is an upstream transcription factor of *LpNOL*. (A) Y1H assay. (B) Relative expression of *LpCbDR1* in root, stem, crown, as well as young (16 days after leaf emergence, DAE), mature (24 DAE), and late senescent (36 DAE) leaves using RT-qPCR. (C) Subcellular localization of LpCbDR1 in mesophyll protoplasts of perennial ryegrass. Signals of green fluorescence protein (GFP) and DAPI-stained nucleus were shown in green and blue, respectively. BF stands for bright field, and the white bars represent 20 μm. (D) Auto-transactivation assay in yeast. (E) Illustration for the presence of two NAC binding sites (NACBS, CATGTG) on *pLpNOL*. (F) Y1H using truncated *pLpNOL* showing that LpCbDR1 bound to *pLpNOL_-571 ~ −1121_* but not *pLpNOL_-1 ~ −570_*. (G) EMSA showing that LpCbDR1 directly bound *pLpNOL_-761 ~ −791_*, while mutation of the *cis*-element diminished its binding. (H) CUT&Tag-qPCR analysis showing higher enrichment of *pLpNOL_-883 ~ −721_* containing the binding site than another fragment away from the site.

To identify additional genetic components involved in the regulation of drought-induced leaf chlorosis, we conducted a yeast one-hybrid (Y1H) library screening assay to identify the upstream transcription factors of *LpNOL*. The promoter of *LpNOL* (abbreviated as *pLpNOL*) was used as the bait in the Y1H library screening assay. Among the candidate transcription factors identified by Y1H, one named Chl *b*  Degradation Regulator 1 (LpCbDR1) was confirmed as a positive candidate (Fig. 1A). It is known that the expression of *LpNOL* increased as leaves progressed through senescence and remained low in the other tissue/organs [7]. Similarly, expression levels of *LpCbDR1* were low in roots, stems, crowns, and young leaves at 16 days after leaf emergence (Fig. 1B), while *LpCbDR1* was highly expressed in late senescent leaves (36 days after leaf emergence), showing an approximately 8-fold increase compared to leaves at 24 days after leaf emergence and a 20-fold increase compared to leaves at 16 days after leaf emergence (Fig. 1B).

For the subcellular localization assay, the LpCbDR1–GFP fusion protein was expressed in mesophyll protoplasts of ryegrass. The green fluorescent signal of LpCbDR1–GFP merged with the DAPI-stained nuclear signal (Fig. 1C), demonstrating that LpCbDR1 is a typical nuclear-localized transcription factor. LpCbDR1 has a typical NAC domain at the N′ and a transcriptional regulatory region at the C′ (Fig. S2A and B). LpCbDR1 belongs to the NAC1 subgroup phylogenetically, with its closest ortholog in rice being OMTN3 (LOC_Os12g41680.1) (Fig. S2C). *OMTN3* encodes a drought-responsive transcriptional factor that negatively regulates drought tolerance in rice [24]. To test whether LpCbDR1 is a transcriptional activator or repressor, we generated a fusion protein of LpCbDR1 with the GAL4 DNA-binding domain (GAL4DBD) and evaluated its transactivation activity using the yeast auto-transactivation assay. The result showed that LpCbDR1 displayed transcriptional activation activity, while the negative control (GUS) did not (Fig. 1D), showing that LpCbDR1 functions as a transcriptional activator.

The direct binding of LpCbDR1 to the promoter of *LpNOL* was further validated by Y1H, electrophoretic mobility shift assay (EMSA) and Cleavage Under Targets and Tagmentation (CUT&Tag)-qPCR assays (Fig. 1E–H). Two potential NAC binding sites (NACBS, CATGTG) were found on *pLpNOL_-778 ~ −773_* and *pLpNOL_-507 ~ −502_* (Fig. 1E). The Y1H assay with truncated *pLpNOL* fragments showed that LpCbDR1 bound to *pLpNOL_-571 ~ −1121_* but not *pLpNOL_-1 ~ −570_* (Fig. 1F). The protein of LpCbDR1 was expressed in *Escherichia coli* and purified for the EMSA assay that confirmed the direct binding of LpCbDR1 to the *pLpNOL_-791 ~ −761_* probe containing the NACBS *cis*-element (Fig. 1G). The *in vivo* binding of LpCbDR1 to *pLpNOL* was also confirmed using the CUT&Tag-qPCR analysis, showing a preference for the NACBS site on *pLpNOL* (Fig. 1H).

### LpCbDR1 positively regulates natural and dark-induced leaf senescence

**Figure 2:**
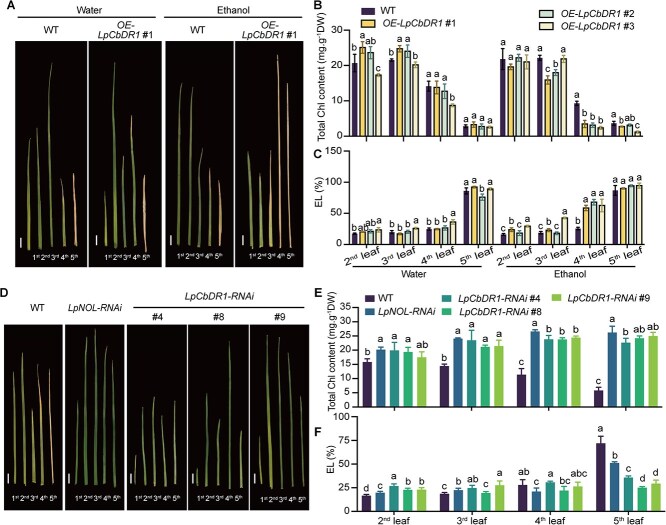
LpCbDR1 positively regulates ryegrass leaf senescence. (A–C) Ethanol-induced *OE-LpCbDR1* transgenic lines had fewer green leaves than the uninduced (treated with water) *OE* lines and the WT plants. (A) Phenotype of the first to fifth leaves from the top, (B) chlorophyll (Chl) contents, and (C) EL rates in wildtype (WT) and *OE-LpCbDR1* sprayed with water or ethanol, respectively. (D–F) *LpCbDR1-RNAi* transgenic lines delayed leaf senescence. (D) Phenotype, (E) Chl content and (F) EL rates. For Chl content and EL measurements in (B, C, E, F), the second to fifth leaves of four plants were harvested and pooled to form one biological replicate. Three biological replicates were included in this experiment. White bars in (A and D) equal to 1 cm. Data in the bar charts are means ± SD (*n* = 3) and the different letters above columns represent significant differences at *P* = 0.05.

To study the molecular function of LpCbDR1, we created transgenic perennial ryegrass plants through *Agrobacterium tumefaciens*-mediated genetic transformation. Initial attempts to overexpress *LpCbDR1* using the maize *Ubiquitin* promoter failed, likely due to its strong transactivating effect on *LpNOL* causing Chl degradation. Therefore, we employed an ethanol-inducible system to regulate *LpCbDR1* overexpression. Two ethanol-inducible *LpCbDR1* overexpression lines (*OE-LpCbDR1*) were selected for further analysis, showing 2.0- to 5.6-fold higher expression levels after ethanol induction compared to the WT (Fig. S3A and B). Ethanol treatment did not affect leaf senescence in WT plants, and there were no phenotypic differences between *OE-LpCbDR1* lines and WT without ethanol induction. However, with ethanol induction, *OE-LpCbDR1* lines exhibited more severe leaf senescence in older leaves, with lower chlorophyll content and higher electrolyte leakage (EL) rates, indicating cell membrane damage. Younger leaves showed no significant differences between *OE-LpCbDR1* lines and WT (Fig. 2A–C, Fig. S4A), suggesting that LpCbDR1-mediated leaf senescence is age-dependent.

Stable RNA-interference *LpCbDR1* transgenic ryegrass lines (*LpCbDR1-RNAi*) were also generated with 60% to 90% reduced *LpCbDR1* expression levels (Fig. S3C–E). The expression levels of *LpNOL* were much lower in the *LpCbDR1*–*RNAi* lines than the WT, with some RNAi lines showing undetectable levels by RT-qPCR (Ct values were > 40) (Fig. S3F). These *LpCbDR1*–*RNAi* lines exhibited a ‘stay-green’ phenotype similar to the *LpNOL-RNAi* lines, with more green leaves *per* tiller (5–6 vs 3.5 WT) and much delayed leaf senescence evidenced by lower EL rates and higher Chl contents and photochemical efficiencies (Fv/Fm) in the older leaves (Fig. 2D–F, Fig. S4B and C).

To investigate the role of LpCbDR1 in dark-induced leaf senescence, we subjected detached leaves to 6 days of dark treatment. The overexpression of *LpCbDR1* led to accelerated senescence, as evidenced by faster Chl degradation, reduced Chl *b*/Chl *a* ratio, decreased Fv/Fm, and increased expression of Chl catabolic genes compared to the WT (Fig. S5). Conversely, the *LpCbDR1-RNAi* exhibited much delayed dark-induced leaf senescence rates: all three lines showed significantly higher total Chl contents, higher Chl *b*/Chl *a* ratios, lower EL values, and lower expression levels of Chl catabolic genes (e.g. *LpNOL*, *LpNYC1*, and *LpPPH*) after 6 days after dark treatment (DAD) (Fig. S6).

### Suppression of *LpCbDR1* delays drought-induced leaf senescence

**Figure 3:**
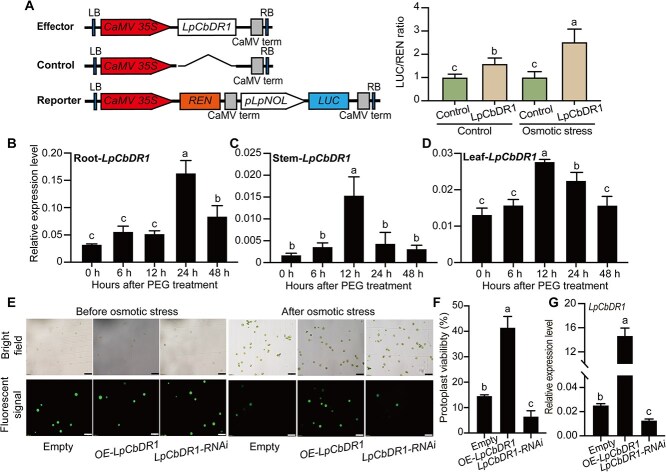
LpCbDR1 is a drought-inducible transcription factor. (A) *In planta* transactivation assay by co-expressing *35S::LpCbDR1* and *pLpNOL::LUC* in untreated and osmotic stress-treated leaves of *N. benthamiana*. The *35S::REN* was used as the internal reporter control; and the empty effector vector was used as the negative control. (B–D) Relative expression of *LpCbDR1* in roots, stems, and leaves after PEG6000 treatment. (E–G) Overexpression or suppression of *LpCbDR1* significantly affected osmotic stress tolerance of ryegrass mesophyll protoplasts. Microscopic images in (E and F) show overall survival rates of protoplasts after osmotic stress at 100 mM mannitol for 30 minutes. Living protoplasts were stained with FDA to discriminate from the dead ones. Empty vector (EV) was used as the negative control. (G) Relative expression of *LpCbDR1* in protoplasts. Observation of each optical field was counted as one replicate with >30 protoplasts in each optical field. The means of protoplast viability (%) were from three optical fields (three replicates). Bars in (E) indicates 50 μm. Data in the bar charts are means ± SD (*n* = 3) and the different letters above columns represent significant differences at *P* = 0.05.

*In planta* transcriptional activation assays demonstrated that co-expression of *LpCbDR1* and *pLpNOL::LUC* resulted in significantly higher LUC/REN values compared to the empty vector control (Fig. 3A). Additionally, 16-hour osmotic stress treatment further enhanced the transactivation effect of *pLpNOL* by LpCbDR1 (Fig. 3A). These results indicate that LpCbDR1 transactivated the expression of *LpNOL* that could be further enhanced by osmotic stress. To verify whether *LpCbDR1* itself is a drought-responsive gene, we analyzed its expression profiles in roots, stems, and leaves of perennial ryegrass following 48 hours of PEG6000 treatment. As shown in Fig. 3B–D, *LpCbDR1* expression was significantly upregulated in all three tissues under PEG6000-induced osmotic stress, confirming its responsiveness to drought. To rapidly investigate the function role of *LpCbDR1* in drought stress response, we performed transiently overexpression and RNA interference (RNAi) of this gene in perennial ryegrass protoplasts. Protoplasts overexpressing or suppressing *LpCbDR1* (with empty vector-expressing protoplasts serving as the control) were treated with osmotic stress. Expression levels of *LpCbDR1* in transfected protoplasts validated the transfection efficiency (Fig. 3E and G). Following osmotic stress, protoplasts overexpressing *LpCbDR1* exhibited a 40% survival rate, significantly higher than the control (13%) (Fig. 3E and F). In contrast, protoplasts with suppressed *LpCbDR1* had a survival rate of only 6%, significantly lower than the control (Fig. 3E and F).

**Figure 4:**
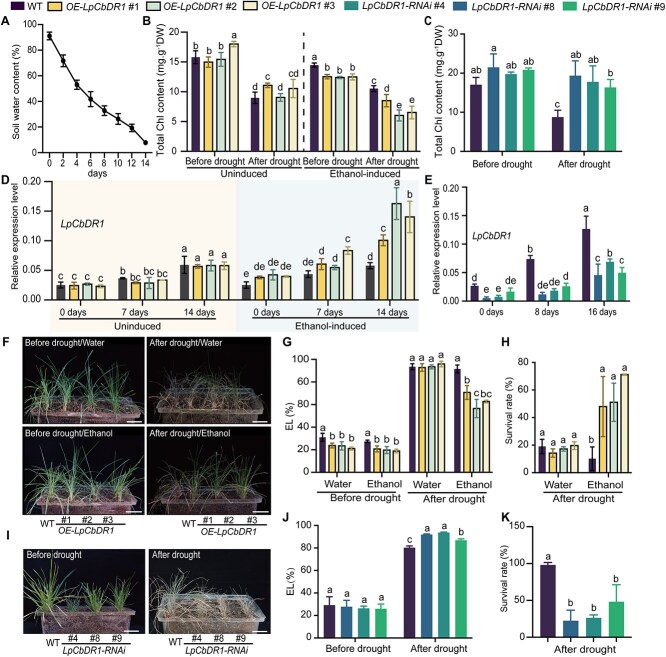
Effects of drought treatment on chlorophyll contents and gene expression in wild-type (WT), *OE*–*LpCbDR1*, and *LpCbDR1*–*RNAi* transgenic lines. (A) Soil water contents during drought treatment. (B and C) Chlorophyll (Chl) contents of WT, *OE* and *RNAi* transgenic lines before and after 14 or 16 days of water-holding for the OE or the RNAi lines, respectively. The *OE-LpCbDR1* were sprayed with 5% ethanol (induced) or water (uninduced) to induce the expression of *LpCbDR1*. (D–G) Relative expression levels of *LpCbDR1*, *LpNOL*, *LpPLA7*, and *LpERF1B* during the drought treatment. The RT-qPCR data are normalized against the reference gene (*LpeIHF4A*). (H–J) Ethanol-induced *OE-LpCbDR1* transgenic lines show improved drought tolerance with less EL and higher survival rates. (K–M) *LpCbDR1-RNAi* transgenic lines had compromised ryegrass drought tolerance. Data in charts are means ± SD (*n* = 3) and the different letters above columns represent significant differences at *P* = 0.05.

To further investigate the role of LpCbDR1 in regulating drought-induced leaf chlorosis, we compared WT, *OE-LpCbDR1*, and *LpCbDR1-RNAi* plants under drought stress conditions (Fig. 4A). Before drought treatment, WT and *OE-LpCbDR1* plants showed similar Chl content and expression level of *LpCbDR1*. However, after prolonged drought treatment, expression level of *LpCbDR1* increased significantly. Ethanol-induced *OE-LpCbDR1* plants exhibited lower Chl contents but higher expression level of *LpCbDR1* compared to WT plants under drought stress (Fig. 4B and D). In contrast, *LpCbDR1-RNAi* plants maintained green leaves with normal Chl contents and lower expression levels of *LpCbDR1* and *LpNOL* (Fig. 4C and E). These findings suggest that LpCbDR1 is essential for drought-induced leaf chlorosis.

### *LpCbDR1* positively regulates drought tolerance

We further investigated the impact of LpCbDR1 on drought tolerance in perennial ryegrass. Without ethanol induction, both WT and *OE-LpCbDR1* lines wilted severely after 16 days of drought treatment, with similar survival rates (16%–20%) after recovery (Fig. 4F–H). However, when induced with ethanol, OE lines showed significantly lower EL rates and higher survival rates (>46%) compared to WT (Fig. 4F–H), indicating enhanced drought tolerance in perennial ryegrass due to *LpCbDR1* overexpression.

Conversely, *LpCbDR1*–*RNAi* lines exhibited compromised drought tolerance (Fig. 4I–K). For example, all *LpCbDR1*–*RNAi* lines had significantly higher EL rates than WT after 14 days of drought treatment (Fig. 4J), and lower survival rates after recovery compared to WT (Fig. 4K). These findings demonstrate that induced overexpression of *LpCbDR1* improves drought tolerance, while RNAi-mediated suppression leads to reduced tolerance in perennial ryegrass.

### Identification of downstream genes of LpCbDR1 using integrated RNA-seq and CUT&Tag analysis

To identify target genes of LpCbDR1 involved in drought tolerance, we conducted integrated transcriptomic and CUT&Tag analyses. Comparative transcriptomic analysis was performed on *OE-LpCbDR1* lines treated with cycloheximide (CHX) alone or with ethanol and CHX. CHX inhibits translation but not transcription [25], minimizing background noise after *LpCbDR1* induction. As shown in Fig. S7, the expression levels of *LpCbDR1* and *LpNOL* in ‘ethanol+CHX’ treated samples were ~3 and 5-fold higher than in those treated with CHX alone, respectively. RNA-seq revealed 3130 differentially expressed genes (DEGs), with metabolic pathways and biosynthesis of secondary metabolites being the most enriched pathways. Gene ontology analysis highlighted roles in oxidoreductase, catalytic, and transferase activities. RT-qPCR validation confirmed the reliability of the RNA-seq results (Fig. S7).

**Figure 5:**
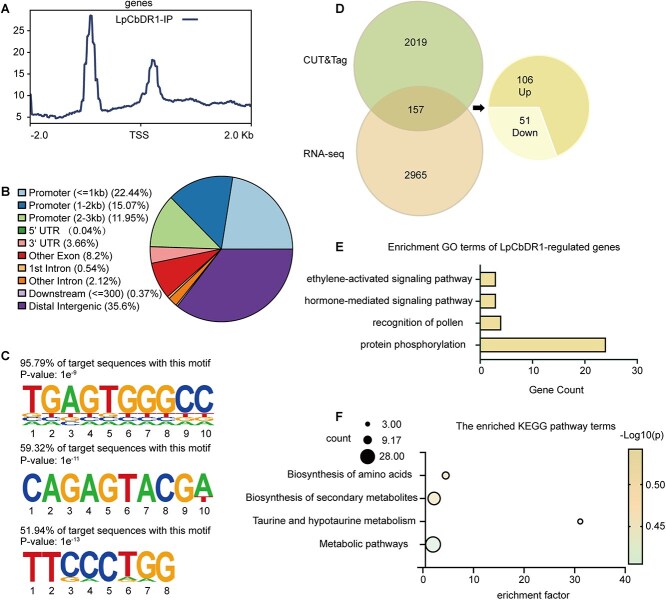
Identification of potential downstream genes of LpCbDR1 using integrated CUT&Tag and RNA-seq analysis. (A) Metaplot of LpCbDR1 binding sites identified by CUT&Tag showing that the LpCbDR1 binding sites centered around the −1 kb upstream of the TSS; (B) Pie chart representing the classification of LpCbDR1 binding sites identified by CUT&Tag; (C) The top three most represented DNA motifs bound by LpCbDR1 present in 95.79%, 59.32%, and 51.94% of target sequences, respectively. (D) Overlapping genes between the RNA-seq DEGs and potential targets identified in the CUT&Tag. (E–F) Top enriched KEGG and GO terms of the 157 overlapping genes.

We conducted CUT&Tag analysis to map the DNA-binding sites of LpCbDR1 (Fig. 5). The analysis revealed that 49.46% of the enriched binding sites were located in promoter regions, with a focus around the −1.0 Kb region of promoters and transcriptional start sites (TSS). Other binding sites included 5′ UTRs (0.04%), 3′ UTRs (3.66%), exons (8.2%), introns (2.66%), downstream sequences (≤ 300 bp, 0.37%), and distal intergenic regions (35.6%). Motif discovery analysis identified three prevalent motifs in the target sequences, accounting for 95.79%, 59.32%, and 51.94% of total motifs, respectively (Fig. 5C; Table S2). The putative NACBS (CATGTG) *cis*-element was present in the predicted DNA-binding motifs but not among the top three prevalent motifs (Table S2). Integration of RNA-seq and CUT&Tag data identified 157 common genes, with 106 up-regulated and 51 down-regulated upon *LpCbDR1* expression (Fig. 5D). As LpCbDR1 is a transcriptional activator, the up-regulated genes are likely its direct targets. As for these up-regulated genes, protein phosphorylation, hormone-mediated signaling pathway, and ethylene-activated signaling pathway were the most enriched GO terms. KEGG analyses showed that the most enriched terms were taurine and hypotaurine metabolism, biosynthesis of amino acids, and biosynthesis of secondary metabolites (Fig. 5E and F).

### *LpPLA7* and *LpERF1B* are downstream target genes of LpCbDR1 involved in drought tolerance

**Figure 6:**
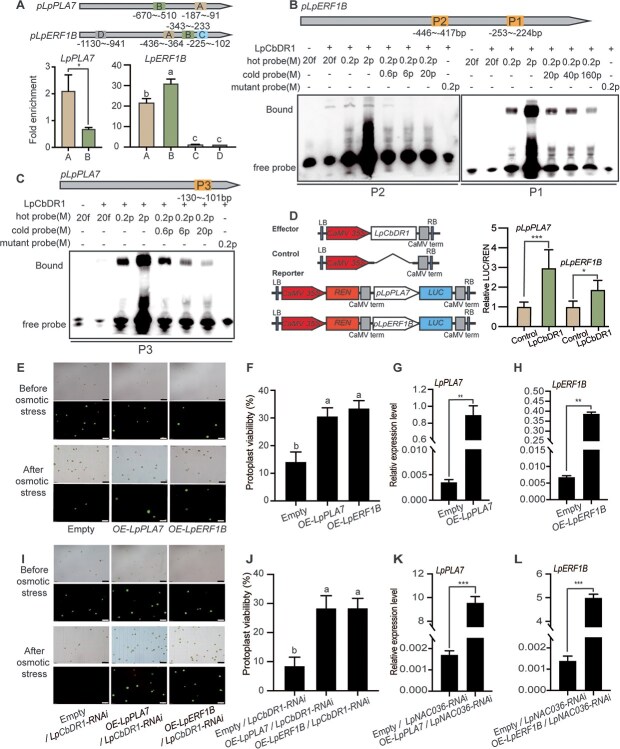
*LpPLA7* and *LpERF1B* are downstream genes of LpCbDR1 involved in drought tolerance. (A) CUT&Tag-qPCR analysis for the binding of LpCbDR1 on *pLpPLA7* and *pLpERF1B*. (B and C) EMSA showing that LpCbDR1 bound *pLpERF1B* and *pLpPLA7* containing the predicted binding sites but not the mutated ones. (D) *In planta* transactivation assay. The *35S::REN* was used as internal control and the empty effector vector as the negative control. (E–H) Overexpression of *LpPLA7* and *LpERF1B* improved protoplast survival rates under the osmotic stress (100 mM mannitol for 30 minutes). (I–L) *LpPLA7* and *LpERF1B* improved osmotic stress tolerance in the background of *LpCbDR1-RNAi* ryegrass. (E&I) Microscopic images showing the fluorescent living protoplasts stained with FDA. (G, H, L) RT-qPCR for gene relative expressions after 16 hours of protoplasts transfection. Protoplasts transfected with empty vectors were used as the negative control. At least 30 protoplasts were counted in one optical field, and the means were from three optical fields. In all charts, different letters represent significant differences at *P* = 0.05, and the bars above the columns represent SD.

Based on the integrated RNA-seq and CUT&Tag analysis, *ethylene-responsive transcription factor 1B-like* (*LpERF1B*, LOC127323795) and *phospholipase A1-II 7-like* (*LpPLA7*, LOC127327743) were identified as two highly up-regulated genes and potential downstream targets of LpCbDR1 (Table S3). The CUT&Tag sequencing analysis predicted specific binding sites for LpCbDR1 on the promoters of *LpPLA7* (*pLpPLA7_–120 ~ − 110_*) and *LpERF1B* (*pLpERF1B_-241 ~ −236_* and *pLpERF1B_-436 ~ −427_*). To validate these predictions, CUT&Tag-qPCR and EMSA analyses were conducted. The results showed significant enrichment of the predicted binding sites on the promoters of *LpPLA7* and *LpERF1B* compared to the control regions (Fig. 6A). EMSA confirmed direct binding of LpCbDR1 to the *cis*-elements on the promoters of *LpPLA7* and *LpERF1B* (Fig. 6B and C). Mutant probes with altered binding sites failed to show binding with LpCbDR1 (Fig. 6B and C). These findings provide strong evidence that LpCbDR1 directly interacted with specific *cis*-elements on the promoters of *LpPLA7* and *LpERF1B*. To assess the activation of *LpPLA7* and *LpERF1B* transcription by LpCbDR1, an *in planta* transactivation assay was conducted by co-expressing *35S::LpCbDR1* with *pLpPLA7::LUC* or *pLpERF1B::LUC* (Fig. 6D). The results indicated a significant increase in transcription of both genes by LpCbDR1 (Fig. 6D).

The phylogenetic tree in Fig. S8 displayed the orthologous genes of *LpPLA7* and *LpERF1B* in rice, wheat (*Triticum aestivum*), and *Brachypodium distachyon*. Functional characterization of orthologous genes of *LpPLA7* was lacking, while ERF1 is a well-known transcription factor for its function in ethylene-signaling regulating abiotic stress-responsive genes [26]. To understand whether expression of *LpPLA7* and *LpERF1B* were responsive to drought stress, we measured their expression in WT, *OE*–*LpCbDR1* and *LpCbDR1*–*RNAi* lines under water-holding treatment. As shown in Fig. S9A–D, expression of both *LpPLA7* and *LpERF1B* increased after 14 days of water-holding (8% soil water content) in WT, and both genes showed higher expression levels observed in *OE*–*LpCbDR1* lines compared to WT, and lower levels in *LpCbDR1*–*RNAi* lines (Fig. S9A–D). Furthermore, under PEG-induced osmotic stress, expression levels of *LpPLA7* increased in roots, stems, and leaves, while LpERF1B only significantly increased in roots within 48 hours of PEG treatment (Fig. S9E–J). These findings supported that both *LpPLA7* and *LpERF1B* were drought-responsive but had different expression patterns.

Furthermore, overexpression of either *LpPLA7* or *LpERF1B* in ryegrass protoplasts resulted in significantly increased expression levels and improved protoplast viabilities under osmotic stress conditions (Fig. 6E–H), suggesting their roles in osmotic stress tolerance. To determine if the stress tolerance conferred by *LpPLA7* or *LpERF1B* depended on LpCbDR1, we overexpressed these genes in the *LpCbDR1*–*RNAi* background. Overexpression of either *LpPLA7* or *LpERF1B* improved protoplast viabilities in the *LpCbDR1*–*RNAi* background, confirming their osmotic stress tolerance functions were not reliant on *LpCbDR1* (Fig. 6I–L). This suggested that *LpPLA7* and *LpERF1B* acted downstream of LpCbDR1 to confer osmotic stress tolerance.

## Discussion

Chl degradation and leaf senescence occur after prolonged drought stress first in older leaves and last in newly emerged leaves. It remains unclear how Chl degradation would affect plant drought tolerance, and additional regulatory genes are to be identified in the coordination of leaf senescence and drought tolerance. In this study, we showed that disrupted Chl *b* degradation by either directly suppressing *LpNOL* or its upstream transcription factor gene (*LpCbDR1*) could abolish the natural, dark- and drought-induced Chl catabolism. Furthermore, LpCbDR1 positively regulates drought tolerance simultaneously by transactivating the expression of *LpERF1B* and *LpPLA7*.

As stated earlier, under drought stress, plants use sophisticated mechanisms to determine whether to remain green. NOL and NYC1 are two Chl *b* reductases that initiate the degradation of Chl catabolism by degrading Chl *b* to 7-hydroxymethyl Chl *a* (HmChl a) [6]. It is interesting to note that both *LpNOL-RNAi* and the *LpCbDR1*-*RNAi* ryegrass plants maintained the stay-green trait with largely unchanged Chl contents during drought stress. On the contrary, Chl contents declined more rapidly in the induced OE lines than in WT. These results suggested that the *LpNOL*-mediated Chl breakdown was indispensable in drought-induced leaf chlorosis. Both *LpCbDR1* and *LpNOL* are among the late drought-responsive genes and are highly expressed in aged leaves [8] [27]. Furthermore, the transcriptional activity of *LpNOL* is greatly enhanced by osmotic stress through the action of transcription factor LpCbDR1 (Fig. 3A). Therefore, these results suggest that ‘LpCbDR1–*LpNOL*’ is an indispensable regulatory module to mandate where (first in aged leaves and then in younger leaves) and when (after prolonged drought stress) ryegrass should initiate leaf senescence in response to drought stress.

Despite the critical role of NOL in initiating Chl *b* degradation, the transcriptional regulation of *NOL* is poorly understood. Little information is available about the upstream transcription factors of the Chl *b* reductase gene *NOL* before. Nevertheless, several transcription factors upstream of the Chl *a* catabolic gene (*SGR*) and another Chl *b* reductase gene (*NYC1*) have been identified and characterized [28–30]. For example, OsNAP accelerates leaf senescence by activating both *SGR* and *NYC1* [31], and LpNAL inhibits leaf senescence by repressing *LpSGR* and *LpNYC1* [30]. BrJUB1 promoted Chl degradation and leaf senescence during the storage of cabbage (*Brassica rapa*) by targeting four Chl catabolic genes [32]. Arabidopsis RD26 was also found targeting multiple stress- and senescence-associated genes, including the Chl *a* catabolic gene (*SGR1*) [16, 18]. However, unlike these transcription factors simultaneously targeting multiple Chl catabolic genes, we did not find other Chl catabolic genes in the CUT&Tag sequencing assay. Likely, *LpNOL* is the only Chl catabolic gene targeted by LpCbDR1.

It is noteworthy that we were unable to obtain *LpCbDR1*'s constitutive overexpression transgenic ryegrass using the maize *ubiquitin* promoter but only be able to obtain transgenic ryegrass using the ethanol inducible expression system [33]. This result suggested that uncontrolled overexpression of *LpCbDR1* might be lethal for perennial ryegrass due to its strong activation effect on *LpNOL*, highlighting the crucial role of the LpCbDR1–*LpNOL* module in fine-tuning the onset and progression of leaf senescence in perennial ryegrass.

The closest ortholog of LpCbDR1 is OMTN3 (LOC_Os12g41680.1) in rice. The known functions of OMTN3 were compromising drought tolerance [24] and immunity against blast fungus (*Magnaporthe oryzae*) [34]. Overexpression of its rice ortholog OMTN3 reduced rice drought resistance [24]. Since *M. oryzae* is not a known pathogen to perennial ryegrass, we focused its regulatory role in senescence and drought responses in this study. However, LpCbDR1 positively regulates ryegrass drought and osmotic stress tolerances, which is contrastingly different from that of OMTN3 in rice. Furthermore, the leaf senescence trait of *OE-OMTN3* transgenic rice was not recorded probably due to little or no altered leaf senescence phenotypic alteration being observed. Unlike *OMTN3* [24], we could not even obtain constitutive *OE-LpCbDR1* ryegrass plants likely due to its strong senescence-promoting effect. According to a genome-wide NAC family analysis, LpCbDR1 (also named as LpNAC036), LpNAC014, and LpNAC064 are the three orthologous genes to OMTN3 [35]. This expansion of these three orthologous genes might have allowed the neofunctionalization of LpCbDR1 during evolution of perennial ryegrass.

*PLA7* and *LpERF1B* were identified as targets genes of LpCbDR1 as well. Phospholipase A (PLA) is a cluster of enzymes that hydrolyze membrane lipids (phospholipids) to generate various cellular mediators (e.g. diacylglycerol, phosphatidic acid, inositol phosphates, 2-acyl-lysophospholipids, and free fatty acids). Besides PLAs, plant phospholipases also include phospholipase D (PLD) and phospholipase C (PLC) according to the site of glycerophospholipid hydrolysis catabolized by these enzymes [36]. Some of these phospholipase-derived products (e.g. phosphatidic acid, inositol phosphates) serve as plant lipid second messengers conferring multifunctional roles in stress signaling [36]. To date, several *PLA*, *PLC*, and *PLD* genes have been found as positive regulators in plant drought stress [37–39]. Phospholipid signaling mediated by *LpPLA7*, one direct target of LpCbDR1, could likely generate lipid-derived second messengers to further interact with other stress-related signaling pathways (e.g. ethylene signaling cascades).

ERF1 is a well-known transcription factor for its function in ethylene-signaling regulating abiotic stress-responsive genes [26]. The Arabidopsis ERF1 is in the downstream of EIN2 and EIN3, acting as an important regulator of ethylene response, including seed germination, root development, and stress (drought and salt) tolerances [40]. Consistent with the known functions of PLAs and the Arabidopsis ERF1, overexpressing *LpPLA7* or *LpERF1* enhanced protoplasts' osmotic stress tolerance. Based on the evidence presented in this manuscript, LpCbDR1 is very likely positioned upstream of ethylene signaling. Integrated CUT&Tag and RNA-seq analyses showed significant enrichment of *ethylene-activated signaling pathway* GO terms among LpCbDR1 direct targets, indicating that ethylene-related genes are transcriptionally regulated by LpCbDR1. Most notably, LpCbDR1 directly binds to and activates *LpERF1B*, an ethylene-responsive gene homologous to *ERF1*, a well-established downstream regulator of EIN2/EIN3 in the canonical ethylene signaling. Functional assays demonstrated that LpERF1B enhances osmotic stress tolerance when overexpressed, and that its expression is positively regulated by LpCbDR1 during drought. Together, these data suggest that LpCbDR1 acts upstream of ethylene-responsive transcriptional outputs, likely amplifying ethylene-mediated stress adaptation. However, it is still unclear how *LpCbDR1* responds to drought and senescence cues and whether other downstream genes of LpCbDR1 are involved in the regulation of leaf senescence and drought tolerance. Further studies on how *LpCbDR1* is integrated in the senescence-associated and drought-responsive signaling pathways (e.g. ABA, ethylene, or other signaling pathways) are to be carried out.

The role of LpCbDR1 in senescence and drought stress regulation places it at the intersection of two important physiological processes, offering new insights into how plants cope with environmental stresses. Given its function in both senescence and stress response, LpCbDR1 could be a valuable candidate for genetic engineering aimed at improving both crop yield and stress resilience. Functions of these downstream genes of LpCbDR1 are worthy of further investigation using stable transgenic ryegrass plants. This dual functionality of *LpCbDR1* positions this transcription factor gene and its downstream genes (e.g. *LpNOL*, *LpPLA7*, and *LpERF1B*) as a promising target for molecular breeding, either through gene editing to develop stay-green cultivars or controlled overexpression to enhance drought resilience.

**Figure 7:**
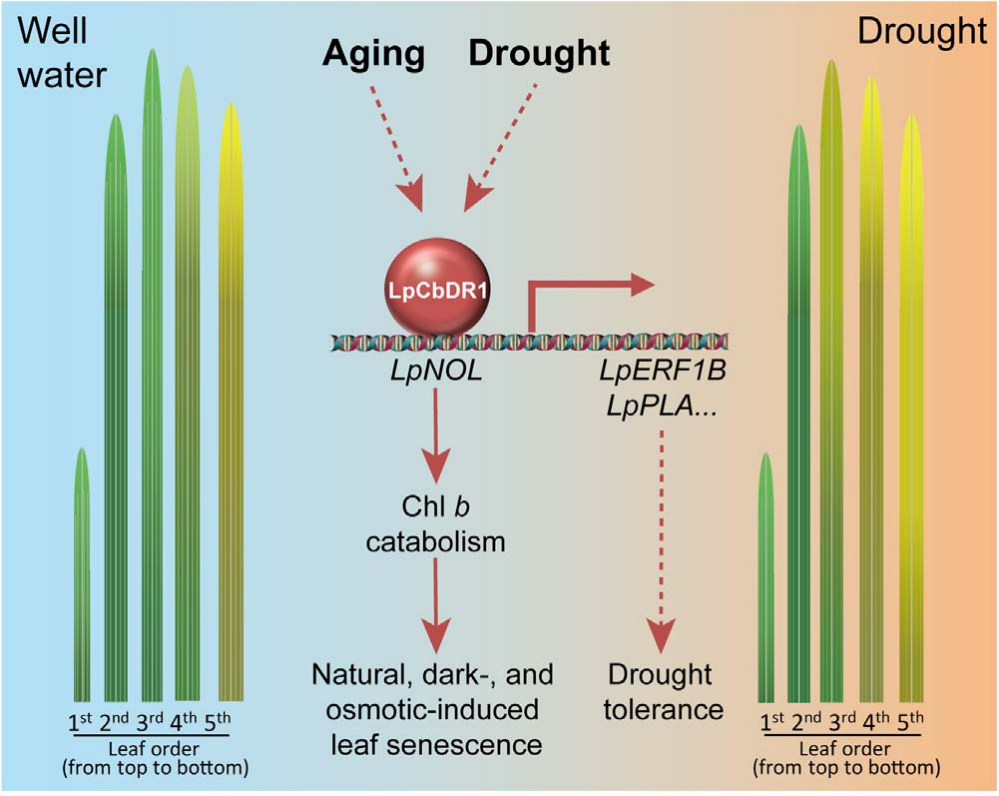
Proposed working model for LpCbDR1 in regulating senescence and drought tolerance. The transcription factor LpCbDR1 is senescence-associated and induced by drought, directly activating the chlorophyll *b* catabolic gene *LpNOL* (evidenced by Y1H, EMSA, CUT&Tag, and *in planta* transactivation assays) to trigger chlorophyll degradation and leaf senescence, starting in older leaves and progressing to younger ones during prolonged drought (transgenic plants, protoplast assays). LpCbDR1 also activates *LpPLA7*, *LpERF1B*, and other genes to enhance drought tolerance (evidenced by EMSA, CUT&Tag, and *in planta* transactivation assay, protoplast assays).

In summary, we showed that blocking Chl *b* degradation by suppressing either *LpNOL* or its upstream transcription factor gene (*LpCbDR1*) diminished drought-induced leaf senescence in perennial ryegrass. LpCbDR1 also regulates plant drought tolerance likely through the regulation of other downstream genes (e.g. *LpPLA7* and *LpERF1*). This work provides insights into the transcriptional regulation of Chl *b* catabolism and drought-induced leaf senescence in an important perennial grass species for better engineering stress-tolerant and stay-green forage and turfgrasses (a working model was proposed in Fig. 7).

## Materials and methods

### Plant materials and growth conditions

Perennial ryegrass cv. ‘Nannong-6#’ was used in this study for gene cloning, genetic transformation, and protoplast-based transient assays. Perennial ryegrass plants were grown in peat: vermiculite mix (3:1, v/v) in a plastic pot (8 cm in diameter and 15 cm in height) and maintained in a greenhouse at 25/20°C with a photoperiod of 14/10 hours (light/dark) and light intensity ~700 μmol·photons·m^−2^·s^−1^ unless otherwise stated for treatments.

### Gene cloning and plasmid construction

The coding sequence (CDS) of *LpCbDR1* was PCR-amplified from cDNA of leaves, cloned into the Gateway entry vector pENTR/D (Invitrogen, Carlsbad, USA), and verified by Sanger sequencing. *LpCbDR1* was recombined into pGADT7, pGBKT7, p2GWF7.0, and pCambia1300-Ubiq-alc [33], respectively. A *LpCbDR1*-specific fragment (Fig. S2) was selected for RNAi. The RNAi fragment was cloned into the pEnD-Kannibal, then recombined to pVT1629 [41] to generate the pVT1629-*LpCbDR1*-*RNAi* construct.

The CDSs of *LpPLA7* and *LpERF1B* were PCR-amplified from leaf cDNA, cloned into the pENTR/D, verified by sequencing, and recombined to pVT1629 [41] for transient overexpression of *LpPLA7* and *LpERF1B* in perennial ryegrass protoplasts. All primers used in this study are provided in Table S1.

### Y1H and auto-transactivation assays in yeast

*LpNOL* has two promoter variants (*pLpNOL-S* and *pLpNOL-L*) differing by a ~ 552-bp deletion in *pLpNOL-S* [42]. Therefore, the longer variant (*pLpNOL-L*) was used in this study. *pLpNOL-L* was cloned into the Y1H bait vector pHis2.1 (Clontech Laboratories, USA) *via* enzyme digestion and ligation to the Sac I/Mlu I sites. The bait vector and the library plasmids were co-transformed with pHis–*pLpNOL* in the yeast strain Y187 (Clontech) individually. Transformants were grown on the SD/-Leu-Trp medium, and positive clones were selected on SD/-Trp-Leu-His plus 10 mM 3-Amino-1,2,4-triazole (3-AT).

For auto-transactivation assays in yeast, pGBKT7-*LpCbDR1* was transformed into the yeast strain Y2H Gold (Clontech) on the SD/-Trp medium. The auto-activation assay was carried out by growing the transformed yeast on the SD/-Trp-His-Ade medium.

### Recombinant protein expression and purification

*LpCbDR1* was subcloned to the pET-30a (+) vector on the restriction sites of EcoRI and PstI to express the LpCbDR1–6 × His fusion protein. The recombinant protein was expressed in *Escherichia coli* strain ‘Rosetta BL21 (DE3)’ induced by 1 mM isopropyl β-d-thiogalactoside (IPTG) at 16°C for 16 hours. *E. coli* was centrifuged down, and 1 mM phenylmethylsulfonyl fluoride (PMSF) was used as a protease inhibitor. The recombinant protein was purified with Ni-IDA resin (Catalog Number: 11402ES60, Cwbio, China) according to the manufacturer’s instructions.

### Electrophoretic mobility shift assay

For the EMSA, two pairs of biotin-5′-end-labeled DNA fragments containing the NAC binding site (CATGTG) were synthesized and annealed to generate double-stranded probes. The cold probes were the unlabeled competitor probes added in 3-, 30-, and 100-fold molar excess. Mutant probes were created by altering the conserved elements to 6 × A. The EMSA was carried out using a Light Shift Chemiluminescent EMSA Kit (Catalog Number: 11402ES60, Thermo Scientific, Waltham, USA). The protein-DNA binding shift was detected using a chemiluminescence imaging system (Vilber Lourmat, Marne la Vallée, France) as suggested by the manufacturer [43].

### Transactivation assay using dual luciferase reporters

To test the transactivation effect of *LpCbDR1* on *pLpNOL*, we generated pGreenII 62-SK-*LpCbDR1* and pGreenII 0800-LUC-*pLpNOL* vectors [44]. The pGreenII 0800-LUC harbors the *35S::REN* within the T-DNA region as an internal reference control [44]. These vectors were transformed into the *Agrobacterium tumefaciens* strain ‘PS105’ (the ‘EHA105’ strain containing pSoup-p19) by freeze–thaw. *Agrobacterium* was resuspended in a buffer (10 mM MES [pH 5.7], 10 mM MgCl_2_, 150 μM Acetosyringone) to a final OD600 of 0.5, then incubated statically for 1 hour. Then, *Agrobacterium* harboring pGreenII 62-SK-*LpCbDR1* or pGreenII 0800-LUC-*pLpNOL* were co-infiltrated into leaves of 4-week-old *Nicotiana benthamiana*. After 56 h, leaf discs were collected with a cork borer. LUC and REN activities were measured using the Dual Luciferase Reporter Gene Assay Kit (catalog: 11402ES60, Yesen, China) on a Tecan Infinite M200 microplate reader. All transfections were performed in triplicate to calculate the LUC/REN.

### Subcellular localization assay

The p2GWF7.0-*LpCbDR1* construct was transformed into ryegrass protoplasts for subcellular localization according to Lei *et al.* [45]. The GFP signal (excitation at 488 nm, scanning at 505–530 nm) and DAPI staining (excitation at 405 nm, scanning at 430 nm) were visualized under a confocal laser scanning microscope (Zeiss LSM780 Exciter). The GFP signal from the empty vector was used as a control.

### RNA extraction, cDNA synthesis, and RT-qPCR

For RNA extraction from leaves, stems, and roots, the Plant RNA Kit (Catalog Number: RK30121, ABclonal Technology Co., Ltd, China) was used according to the manufacturer’s protocol. For osmotic stress, 15% PEG6000 (w/v) was applied onto hydroponically cultured perennial ryegrass. Total leaves were pooled together after 0 to 48 h of PEG treatment for RNA extraction.

For RNA extraction from mesophyll protoplasts, the MolPure® cell RNA kit (Yeasen Biotech. Co., Ltd, Shanghai, China) was used. The first-strand cDNA was synthesized with 1 μg RNA using the PrimeScript RT reagent Kit with gDNA Eraser (Perfect Real Time) (Catalog Number: RK20433, ABclonal). RT-qPCR was conducted using the SYBR Green PCR Master Mix (Catalog Number: RK21220, ABclonal) on a Roche Light Cycler 480 II Real-Time PCR System [46]. *LpeIHF4A* was used as the reference gene [30]. The qPCR primers were designed using the online Real-time PCR primer design tool of GenScript and listed in Table S1. Relative expression levels of target genes were calculated using the 2^−ΔΔCt^ or 2^−ΔCt^ method with the relative expression levels of the reference gene (*LpeIHF4A*).

### *Agrobacterium*-mediated genetic transformation of perennial ryegrass

Transgenic perennial ryegrass plants were generated by *Agrobacterium*-mediated genetic transformation following the protocol described by Xu *et al*. [47]. Briefly, *A. tumefaciens* strain ‘EHA105’ harboring the overexpression (OE) or RNAi vectors at OD600 = 0.6 was used for genetic transformation. Calluses were co-cultured with *Agrobacterium* for 3 d, washed with sterile water, and then transferred to selection medium containing 200 mg∙L^−1^ Timentin and 50 mg∙L^−1^ hygromycin (Roche Diagnostics). After 2 months of culture, the resistant calluses were regenerated and rooted. Hygromycin-resistant plant lines were further confirmed by GUS staining and PCR amplification for the presence of the T-DNA sequence overlapping part of the target gene. Primers used in the PCR were listed in Table S2.

### Dark-induced leaf senescence and drought stress treatment

For dark-induced leaf senescence treatment, fully expanded mature leaves were detached from the plants and sandwiched between two layers of paper towels pre-wetted with 3 mM MES buffer (pH 5.8) at 25°C and incubated in the dark for 6 days [48].

For the drought tolerance test, wild type (WT) and different transgenic lines were grown in the same pot with watering for 2 weeks and then subjected to water withholding for 10 to 16 days. For ethanol-inducible *OE-LpCbDR1* lines, 5% ethanol (water as control) was sprayed onto the plants 2 days prior to the drought treatment and continually sprayed every day after the treatment at a dosage of ~5 ml *per* pot. The survival rates of the drought-treated plants were counted after 2 weeks of watering for recovery. Three replicates of each WT and transgenic line were performed. The soil water content of each pot was measured during the water withholding process using a soil moisture sensor (Model: SN-3000-*-USB, Prsens Environmental Monitoring Inc., China). The survival rate (the number of surviving tillers/total tillers), Chl content, EL, Fv/Fm, MDA, and RWC of each line were measured as described below.

### Chl content measurement

Chl contents of leaves were determined by dimethyl sulfoxide (DMSO) extraction with ~0.1 g of fresh weight samples. Absorbance of the DMSO extract was measured at 645 and 663 nm. Subsequently, the same samples were dried at 65°C for 3 days for dry weight (DW) determination. Chl content was calculated according to Arnon [49].

### EL rates and Fv/Fm measurements

The EL of leaves was evaluated according to Zhang *et al*. [33]. In brief, freshly isolated leaf samples (~0.1 g) were thoroughly rinsed with dH_2_O and then incubated in dH_2_O on a shaker (100 rpm) for 24 hours at room temperature. Their initial conductivity (C_i_) values were measured using a calibrated conductivity meter. Samples were then autoclaved at 121°C for 20 minutes for complete electrolyte release to measure the maximum conductivity (C_max_) values. Relative EL was calculated as: EL (%) = (C_i_/C_max_) × 100.The maximum photochemical efficiency of PSII (Fv/Fm) of leaves was measured after 30 minutes dark acclimation using a fluorescence induction monitor (Bioscientific Ltd, Herts, United Kingdom).

### Perennial ryegrass mesophyll protoplast isolation and transfection

The fully expanded leaves (the second leaves from the top) were taken from robustly grown ryegrass plants for the protoplast isolation and transfection following the protocol described by Lei *et al*. [45]. The protoplast viability was ~83% after enzyme digestion for protoplast isolation. Then, the broken cell debris was removed using centrifugation. The protoplast integrity was examined again under a light microscope. After removal of the cell debris by centrifugation, the viability of isolated protoplasts was checked under a light microscope. (Olympus Model BX53, Tokyo, Japan). Ten-microgram plasmids were used for protoplast transfection. Successfully transfected mesophyll protoplasts with transfection efficiency over 90% were used in the CUT&Tag and osmotic stress treatment.

The osmotic stress treatment on transfected mesophyll protoplasts was performed following the ‘protoplast-based rapid stress regulatory gene identification assay’ [45]. In brief, ryegrass protoplasts transfected with 10 μg plasmids of p2GWF7.0 empty vector, p2GWF7.0-*LpCbDR1*, p2GWF7.0-*LpCbDR1 × RNAi*, p2GWF7.0-*LpPLA7*, and p2GWF7.0-*LpERF1B* were incubated for 16 h at 25°C. Then, 5 μl of 1 M mannitol was added to 45 μl protoplast suspension to a final concentration of 100 mM. After 30 minutes incubation at room temperature, 5 μl of 10 μM fluorescein diacetate (FDA) solution was added to 5 μl protoplasts, gently mixed, and incubated for 2 min. Protoplasts were then photographed under a microscope (Olympus Model BX53). Protoplasts with a fluorescent signal were counted as living protoplasts. The number of viable and total protoplasts was counted for the calculation of protoplast viability (%). Observation of each optical field was counted as one replicate, with >30 protoplasts counted in each optical field. The means of protoplast viability (%) were from three optical fields (three replicates).

### RNA-sequencing

The transgenic ryegrass lines harboring *LpCbDR1* driven under an ethanol-inducible promoter were sprayed with either 10 μM CHX (a protein synthesis inhibitor) alone as a control (CHX) or induced with CHX plus 5% ethanol (eth). The surfactant, Silwet-77 (final concentration of 0.015%), was added to these solutions to help the sprays adhere evenly to the leaf surface. Their third leaves from the top were pooled for RNA-seq after 6 hours of induction by ethanol.

The RNA-seq was outsourced to Gene Denovo Inc. (Guangzhou, China) for library construction and sequencing using the Illumina Novaseq X Plus platform. The comparative transcriptomic analysis was performed on the Omicsshare platform (Gene Denovo). In brief, paired-end clean reads were mapped to the perennial reference genome (GenBank: JAEVFB000000000) [42] using HISAT2 2.1.0 with default parameters. For each transcript, TPM (Transcripts *Per* Kilobase of exon model *per* Million mapped reads) values were calculated to quantify its expression abundance. Differential expression analysis was performed by DESeq2 software between the ‘CHX’-treated samples and the ‘CHX + ethanol’-treated samples. DEGs were identified with the parameters of false discovery rate (FDR) below 0.05 and absolute fold change ≥2. Using the DEGs, Gene Ontology (GO) and KEGG enrichment analyses were performed using the Gene Ontology Resource database and the KEGG Compound database, respectively.

### CUT&tag

Ryegrass mesophyll protoplasts transfected with 10 μg plasmids of p2GWF7.0-*LpCbDR1* were placed in an incubator for 16 hours at 25°C to allow gene expression. Then, CUT&Tag was carried out using a Kit (catalog number: N259-YH01-01B, Novoprotein, China) and anti-GFP antibody (catalog number: ab290, Abcam, UK) following the manufacturer's manual. For each sample, 1 μg of GFP antibody was added, followed by incubation at 4°C for 8 to 12 hours. The resultant DNA was sent to Nanjing BioBay Inc. (Nanjing, China) for the CUT&Tag-seq library construction and sequenced using the Illumina Novaseq 6000 platform.

For CUT&Tag-seq data analysis, clean CUT&Tag-seq reads were mapped to the latest perennial ryegrass reference genome [50]. CUT&Tag-seq peaks were identified by MACS2 (version 2.1.1) using default parameters (*P* < 0.05). The gene closest to the LpCbDR1 binding sites was defined as a putative binding gene when searched with Peak Annotator. The enriched peaks were visualized with Integrative Genomics Viewer (IGV, version 2.8.2). Motifs were discovered using Homer version 3.

For CUT&Tag-qPCR, the resultant DNA from CUT&Tag was used as a template for qPCR with primers listed in Table S1. Enrichment folds were calculated using Input (without GFP antibody) as the control.

### Statistical analysis

For statistical analysis, data were analyzed using SPSS software (IBM SPSS software, USA). Student’s *t*-test was used to compare two specific groups, and Duncan's test was used to compare three or more groups. A minimum of three replicates was used for all quantitative tests. Data were presented as mean ± standard deviation (SD).

### Accession numbers

NCBI GenBank accession numbers of the proteins used in this study are LpCbDR1 (XM_051328527.2), LpNOL (KX686493), LpPLA7 (XP_051210499.1), and LpERF1B (XP_051207879.1).

## Supplementary Material

Web_Material_uhag093

## Data Availability

The raw sequencing data generated during this study are publicly available in GenBank (SRA No.: SUB15346059).
