# Supplementary material for: LpCbDR1 regulates leaf senescence and drought tolerance by activating the chlorophyll b reductase gene and stress-related genes in perennial ryegrass
Source: Hortic Res. 2026 Mar 9;13(7):uhag093. doi: 10.1093/hr/uhag093 (PMC13305888; doi:10.1093/hr/uhag093)
Supplement: Web_Material_uhag093 [file web_material_uhag093.zip › S-Figures.docx]

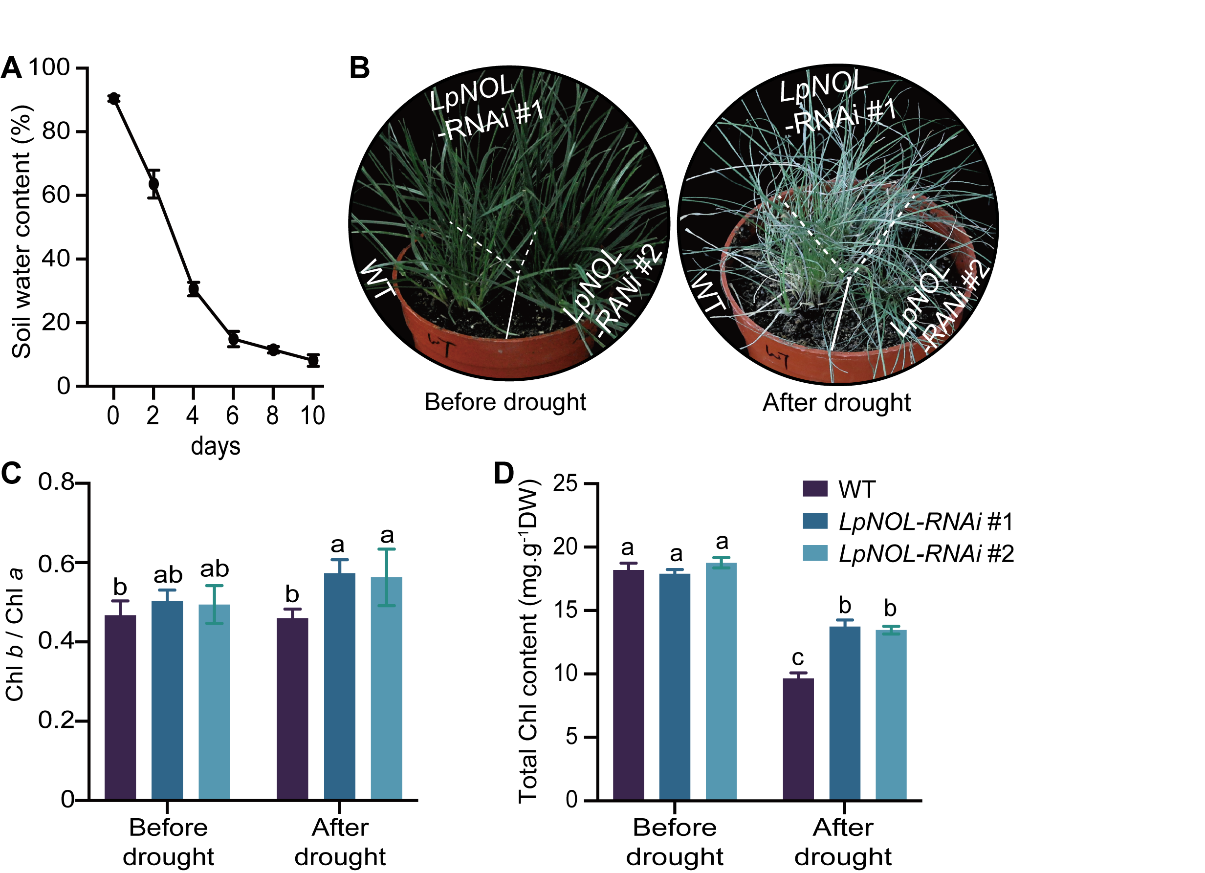


**Figure S1.** Suppression of *LpNOL* delayed drought-induced chlorosis. (A) Soil water content during the drought treatment. (B) Phenotype of the wild type (WT) and the *LpNOL-RNAi* lines before and after 10 d of water withholding. (C-D) Chlorophyll contents and Chl *b* : Chl *a* ratio of WT and *LpNOL-RNAi* lines before and after the drought stress. Data in the line and bar charts are means ± SD (n = 3). The different letters above columns represent significant differences at *P* = 0.05.

**
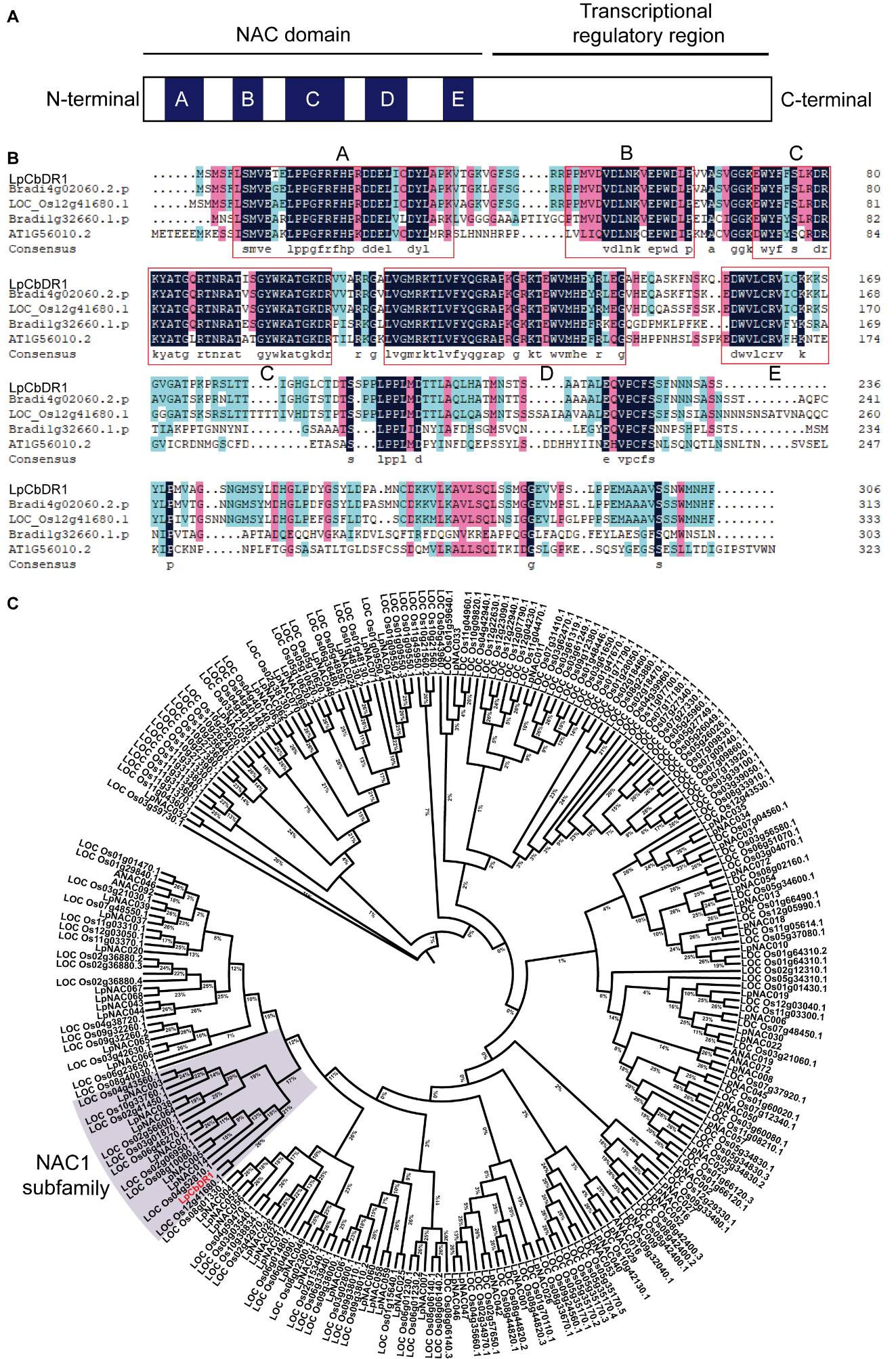
**

**Figure S2.** Multiple sequence alignment and phylogenetic tree analyses of LpCbDR1. (A) Protein domains of LpCbDR1 showing the conserved NAC domain at the N terminus and the transcriptional regulatory region at the C terminus. (B) Sequence analysis of LpCbDR1 homologs in *Brachypodium distachyon*, rice (*Oryza sativa*), and *Arabidopsis thaliana*. (C) Neighbor-joining (NJ) phylogenetic tree constructed using the MEGA11 software with NAC family proteins in perennial ryegrass and rice.


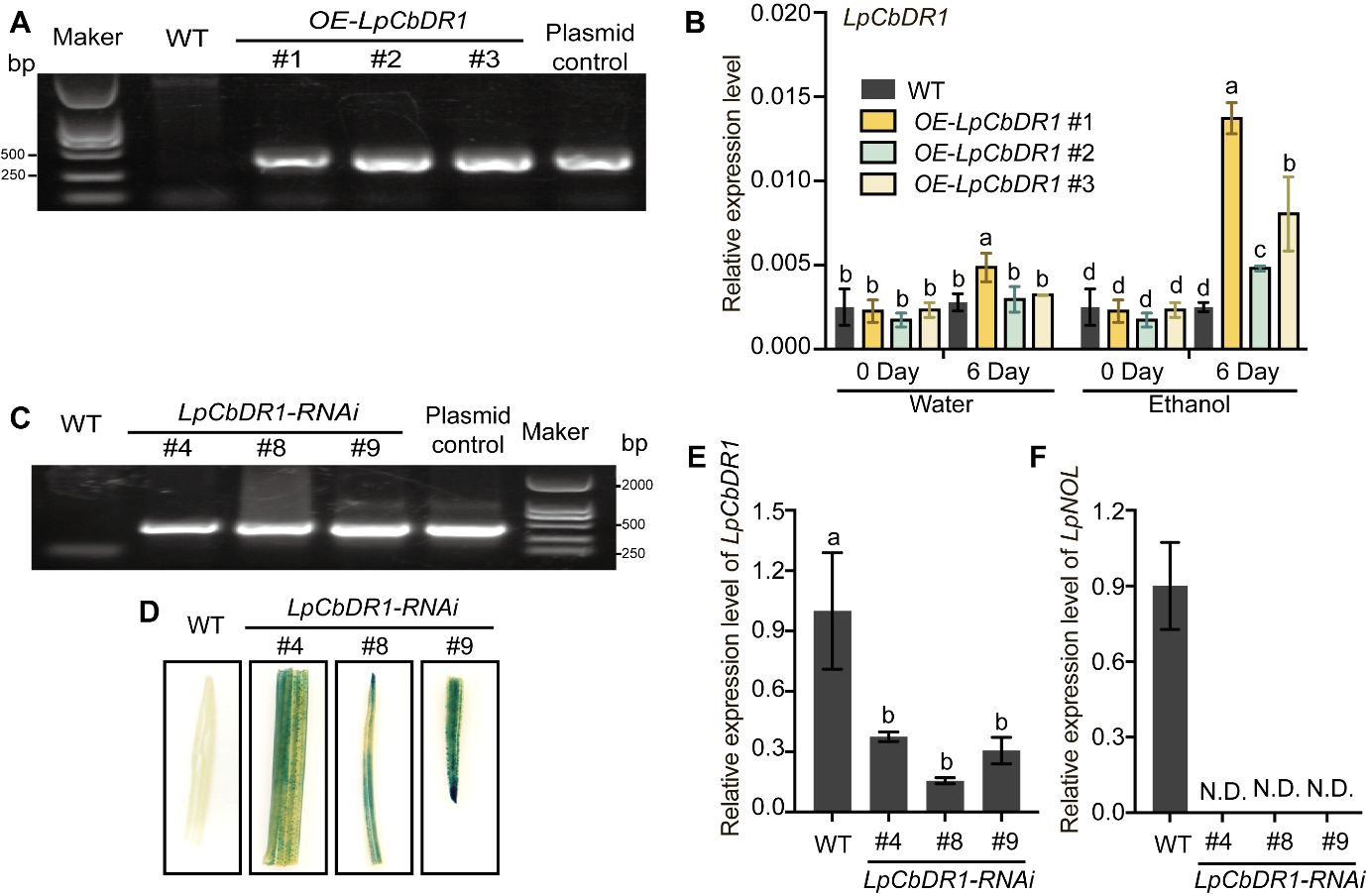


**Figure S3.** Verification of ethanol inducible overexpression (*OE-LpCbDR1*) and *LpCbDR1-RNAi* transgenic ryegrass. (A) PCR detection for the presence of T-DNA. (B) RT-qPCR analysis of ethanol-inducible expression of *LpCbDR1* in wild type (WT) and *OE-LpCbDR1* lines. (C) PCR detection for the presence of T-DNA. (D) GUS staining. (E) Relative expression of *LpCbDR1* and its downstream gene, *LpNOL*, in WT and *LpCbDR1-RNAi* lines. Data in (B, C&D) are means ± SD (n = 3). The different letters above columns represent statistically significant differences at *P* = 0.05.

**
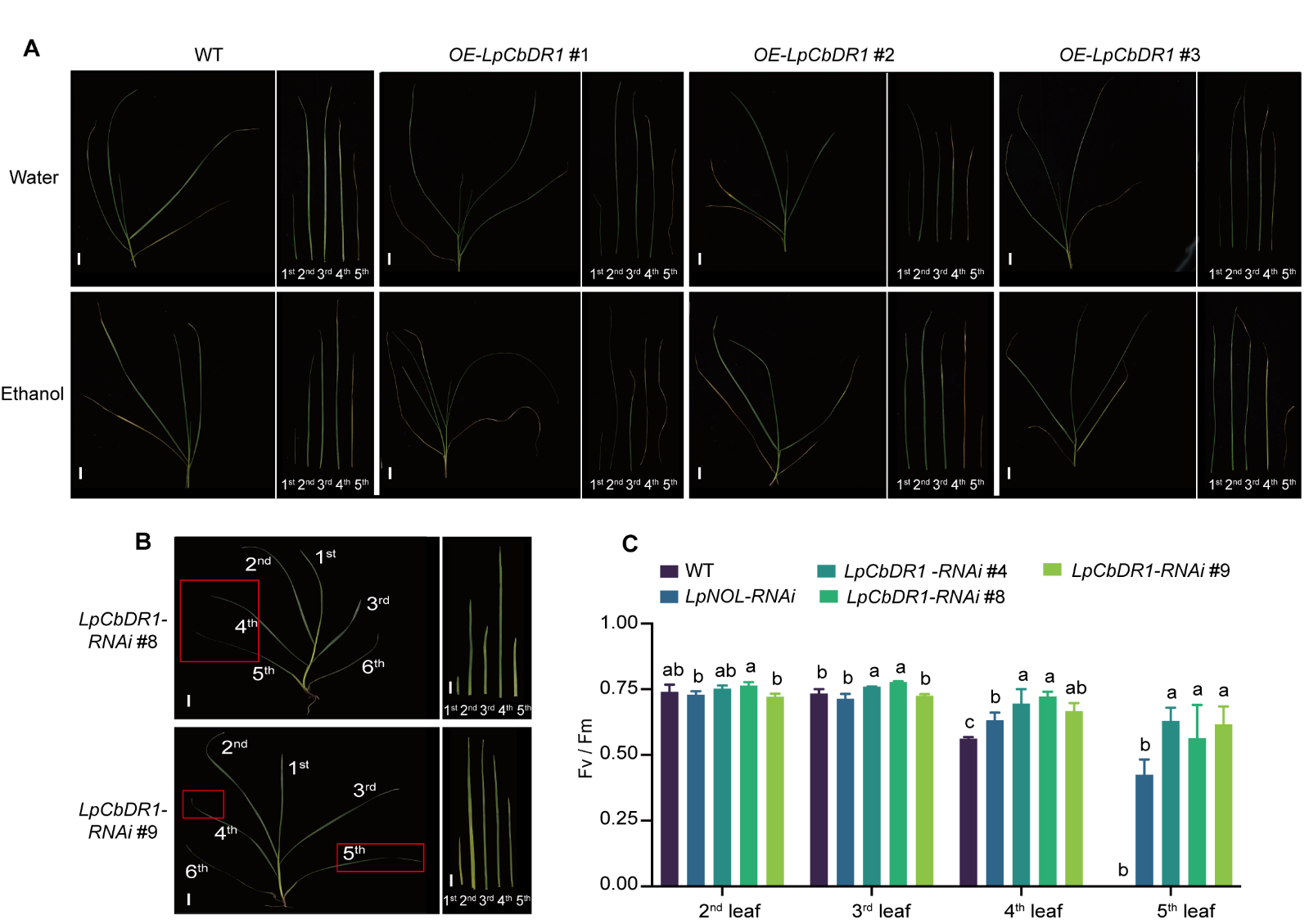
Figure S4.** Phenotypes of the *OE-LpCbDR1* and *LpCbDR1-RNAi* lines. (A) After the inducible overexpression of *LpCbDR1*, the *OE-LpCbDR1* lines appeared to have fewer green leaf numbers than WT. Ethanol (5%) was sprayed onto the WT and *OE* plants. (B) *LpCbDR1-RNAi* lines had more green leaf numbers than the WT. (C) Fv/Fm of different leaves from the top of each tiller in *LpCbDR1-RNAi* lines. Data in (C) are means ± SD (n = 3) and the different letters above columns represent statistically significant differences at *P* = 0.05.


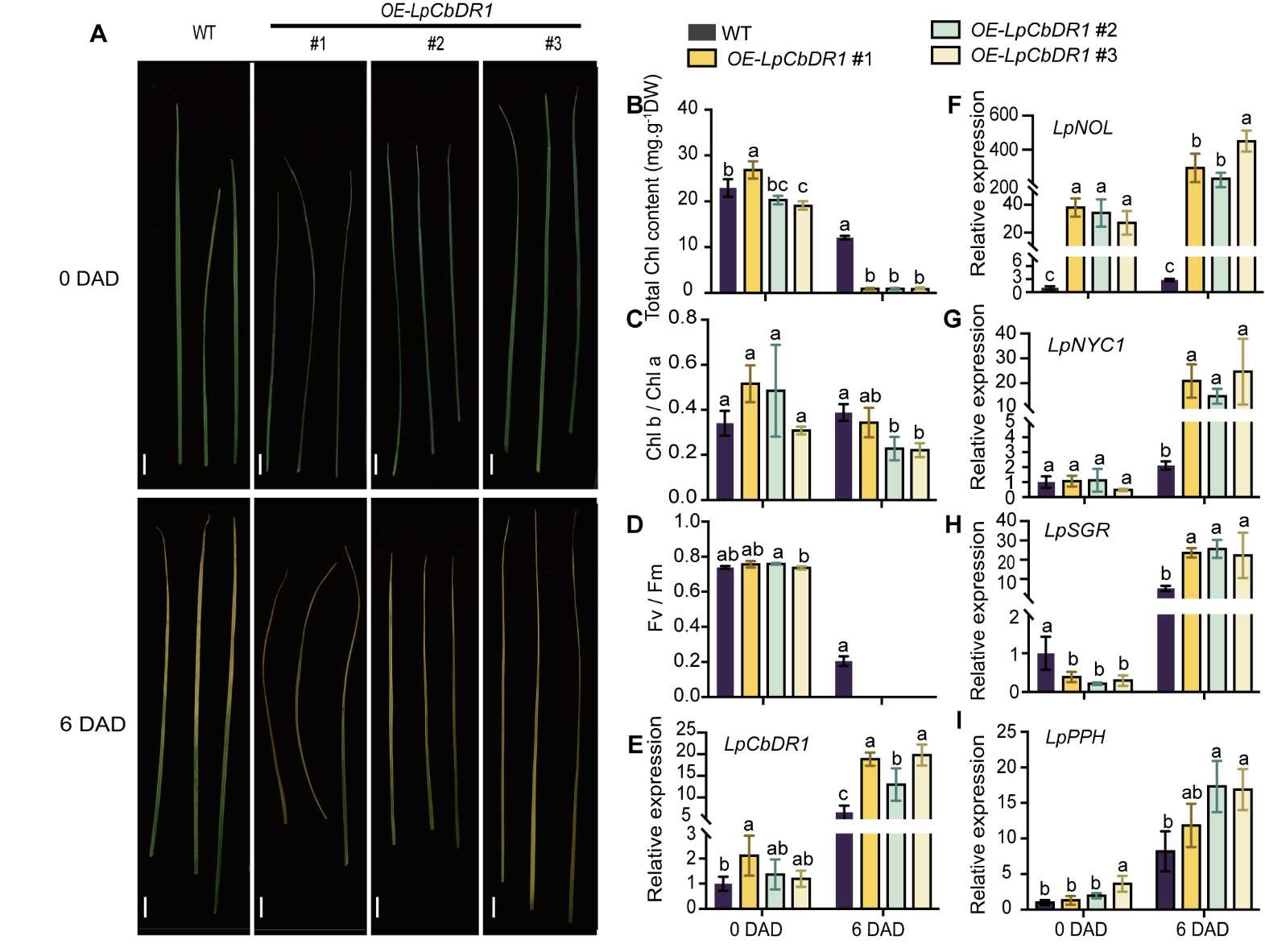


**Figure S5.** Inducible overexpression of *LpCbDR1* accelerated dark-induced leaf senescence. (A) Phenotype of the leaves before and after dark incubation (DAD: days after dark treatment). White bars equal to 1cm. (B-D) Total Chl contents, Chl*b*/Chl*a* ratios, and Fv/Fm of the treated leaves. (E-I) Relative expression levels of *LpCbDR1* and Chl catabolic genes (e.g., *LpNOL*, *LpNYC1*, *LpPPH*, and *LpSGR*) using RT-qPCR. Data in (B-J) are means ± SD (n = 3) and the different letters above columns represent statistically significant differences at *P* = 0.05.


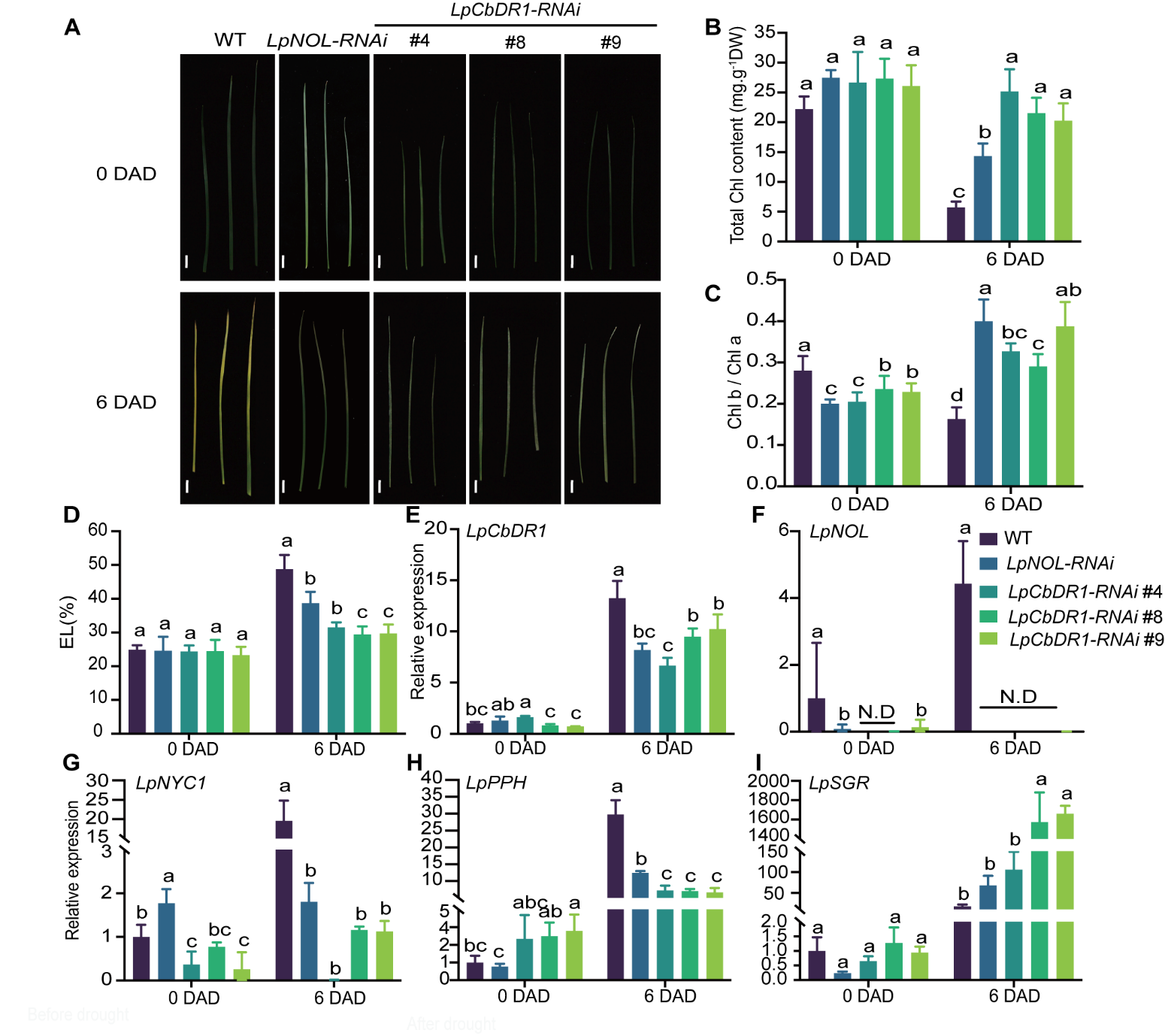


**Figure S6.** Constitutive suppression of *LpCbDR1* delayed dark-induced leaf senescence. (A) Phenotypes of WT, *LpNOL-RNAi*, and *LpCbDR1-RNAi* before and after dark incubation (DAD: days after dark treatment). White bars equal to 1cm. (B-D) Total Chl contents, Chl*b*/Chl*a* ratios, and electrolyte leakage (EL) rates of the treated leaves. (E–I) Relative expression levels of *LpCbDR1* and chlorophyll catabolic genes (e.g., *LpNOL*, *LpNYC1*, *LpPPH*, and *LpSGR*) using RT-qPCR. Data in (B-J) are means ± SD (n = 3) and the different letters above columns represent statistically significant differences at *P* = 0.05.

**
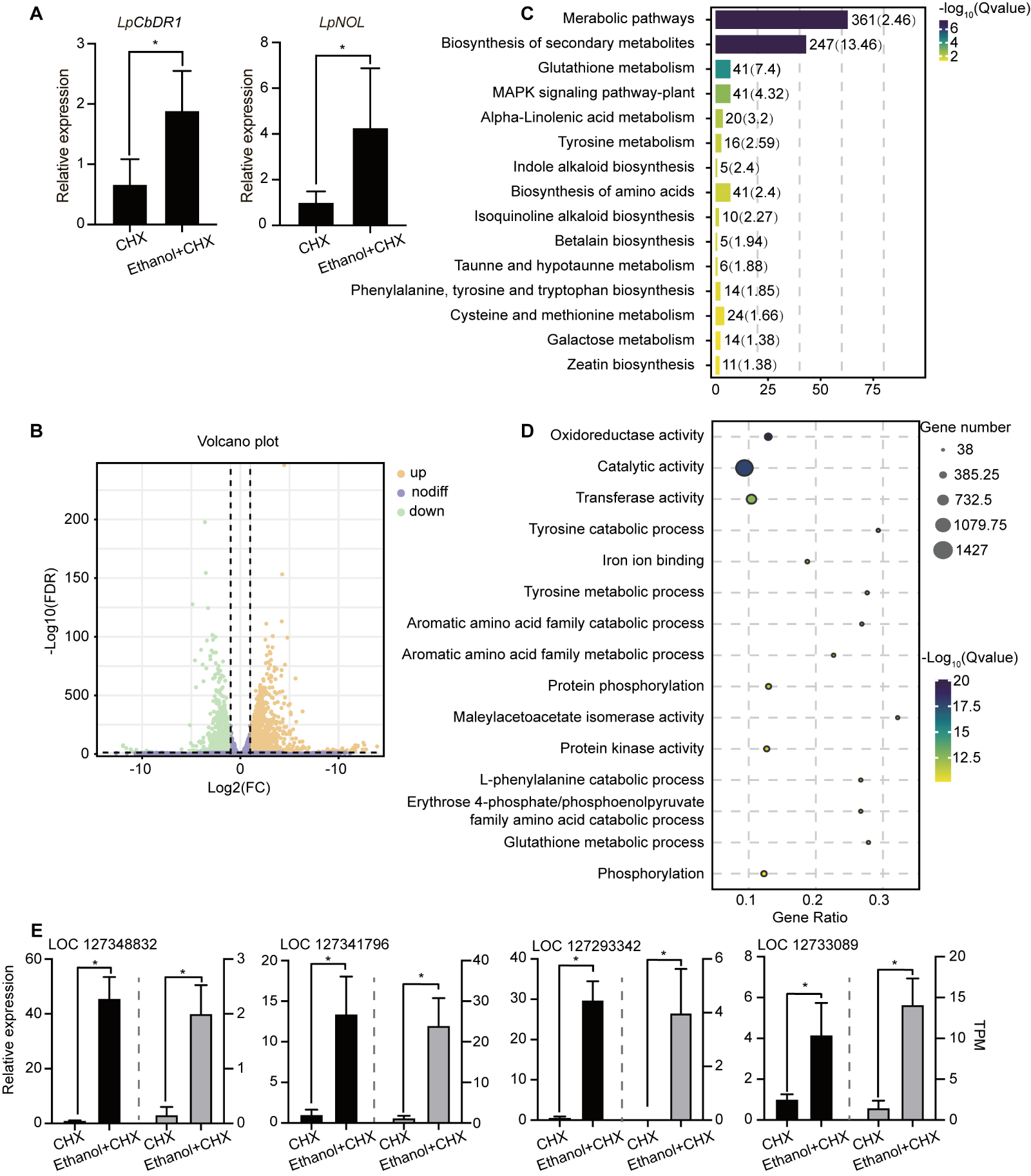
Figure S7.** Transcriptomic alterations due to the inducible expression of *LpCbDR1*. The *OE-LpCbDR1* transgenic lines were sprayed with either 10 μM cycloheximide (CHX, a protein synthesis inhibitor) alone as a control (CHX) or induced with CHX plus 5% ethanol (Ethanol + CHX) for the overexpression of *LpCbDR1*. (A) Relative expression levels of *LpCbDR1* and *LpNOL* using RT-qPCR of the samples used for RNA-seq. (B) Volcano plots revealing the differentially expressed genes (DEGs) due to inducible expression of *LpCbDR1*. (C) Top enriched KEGG pathways. (D) Top enriched GO terms. (E) Validation of the RNA-seq using qRT-PCR. Relative expression values from RT-qPCR and TPM values of the RNA-seq analysis of four DEGs were shown. Data in (A&E) are means ± SD (n = 3) and the different letters above columns represent statistically significant differences at *P* = 0.05.


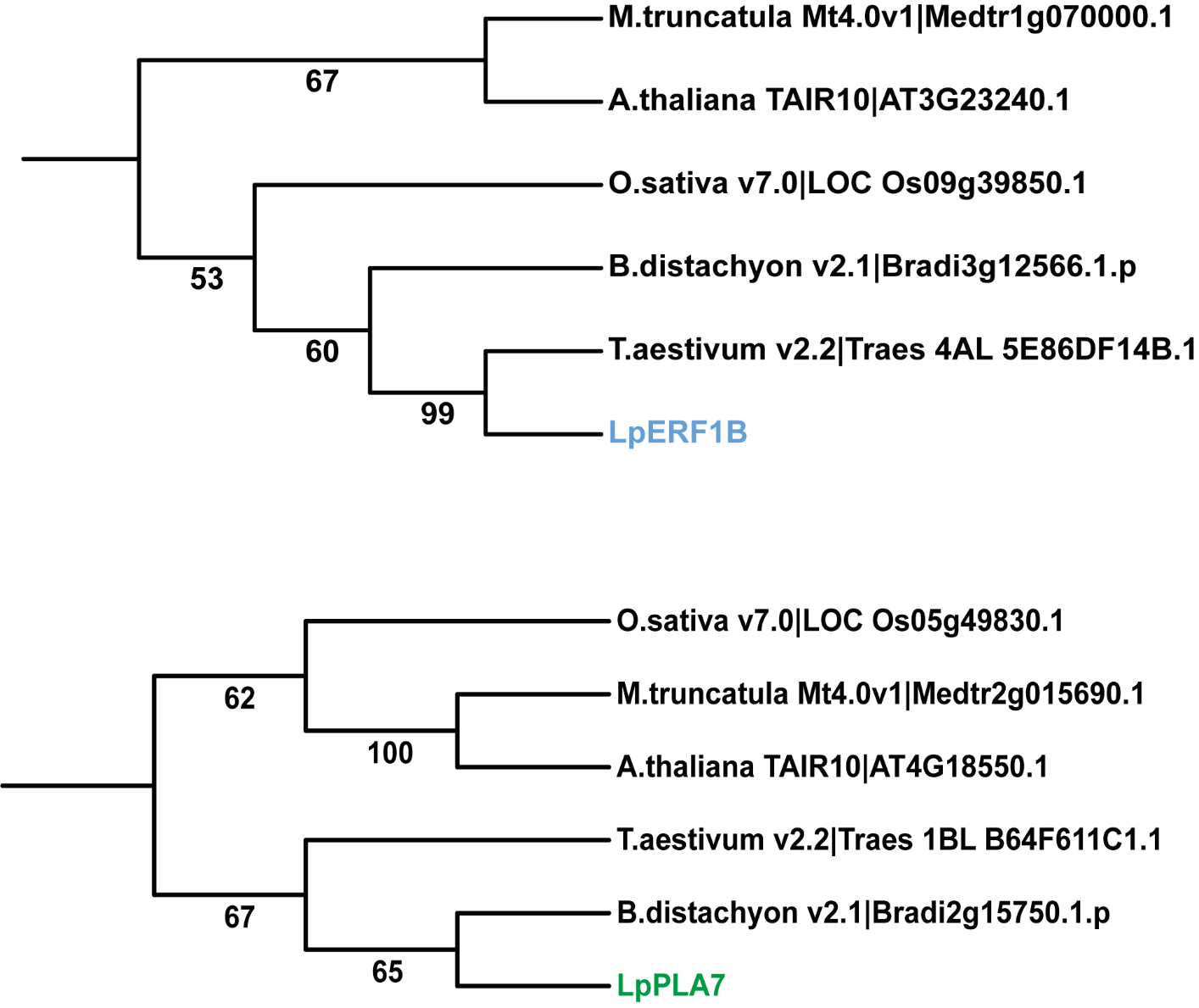


**Figure S8**. Phylogenetic tress of LpPLA7 and LpERF1B with their orthologous proteins in *Brachypodium distachyon*, *Oryza sativa*, *Triticum aestivum*, *Medicago truncatula*, and *Arabidopsis thaliana*.


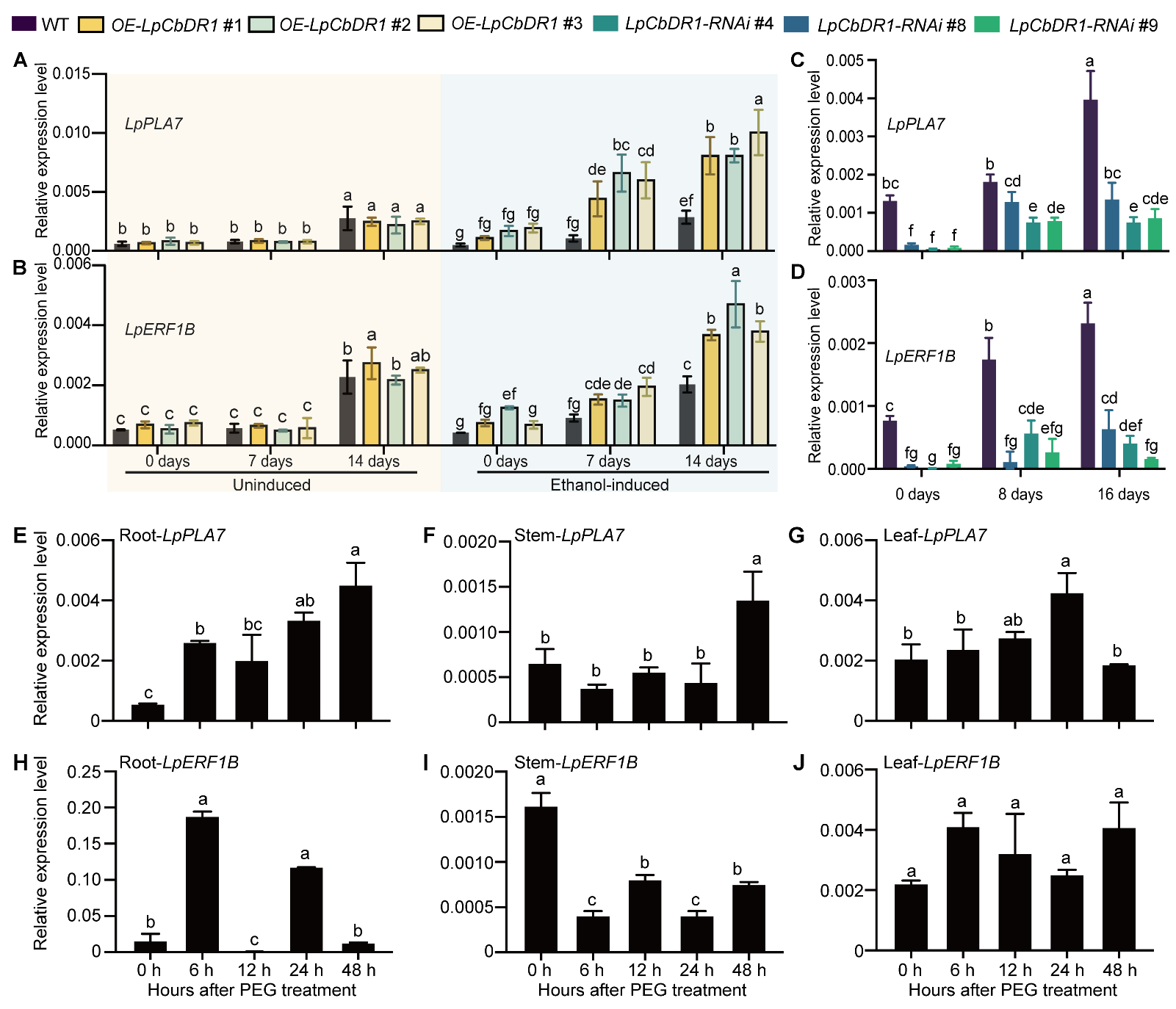
**Figure S9**. Relative expression levels of *LpPLA7*, and *LpERF1B* during drought and osmotic stress treatments. (A-D) Relative expression of *LpPLA7*, and *LpERF1B* in WT, OE-*LpCbDR1*, and *LpCbDR1-*RNAi transgenic lines during the drought/water-holding treatment using RT-qPCR normalized against the reference gene (*LpeIHF4A*). (E-J) Relative expression in roots, stems and leaves after PEG6000 treatment. Data in the bar charts are means ± SD (n = 3) and the different letters above columns represent significant differences at *P* = 0.05.

**Table S1. Primers and probes used in the study.**

| Probes used in EMSA | Sequence (5' to 3') | Notes |
| --- | --- | --- |
| *LpNOL*-hot probe-F | CACGGCATGTCACATGTGCCAACACTGGAT | Probes used in EMSA |
| *LpNOL*-hot probe-R | ATCCAGTGTTGGCACATGTGACATGCCGTG | Probes used in EMSA |
| *LpNOL*-mutant probe-F | CACGGCATGTCAAAAAAACCAACACTGGAT | Probes used in EMSA |
| *LpNOL*-mutant probe-R | ATCCAGTGTTGGTTTTTTTGACATGCCGTG | Probes used in EMSA |
| *LpERF1B*-hot probe3-F | GCATAGTGATGGAGAGGGAGTTTGAGGATA | Probes used in EMSA |
| *LpERF1B-*hot probe3-R | TATCCTCAAACTCCCTCTCCATCACTATGC | Probes used in EMSA |
| *LpERF1B*-hot probe2-F | CATGCCCGTCTGTGTATCCTATTCGTTGA | Probes used in EMSA |
| *LpERF1B*-hot probe2-R | TCAACGAATAGGATACACAGACGGGCATG | Probes used in EMSA |
| *LpPLA*-hot probe1-F | GGTATAAATATCAGTGCGCAAGATACTAGC | Probes used in EMSA |
| *LpPLA*-hot probe1-R | GCTAGTATCTTGCGCACTGATATTTATACC | Probes used in EMSA |
|  |  |  |
| Primers used in CUT&Tag-qPCR | **Sequence (5' to 3')** |  |
| *LpNOL*-AF | TACGACGGGCTATTTGCTGA | Primers used in CUT&Tag-qPCR |
| *LpNOL*-AR | CGCTGAGATAACGAGCCAAG | Primers used in CUT&Tag-qPCR |
| *LpNOL*-BF | GGTTACATGGGCATGTGCAG | Primers used in CUT&Tag-qPCR |
| *LpNOL*-BR | TCTAGGCCAGAAACGTACCC | Primers used in CUT&Tag-qPCR |
| *LpPLA*-AF | CGTTTCGGTCAGACAAGACC | Primers used in CUT&Tag-qPCR |
| *LpPLA*-AR | GGCTAGTATCTTGCGCACTG | Primers used in CUT&Tag-qPCR |
| *LpPLA*-BF | GCCGATACCCGATAGACGAT | Primers used in CUT&Tag-qPCR |
| *LpPLA*-BR | CTTGTTGTCGAGTGGTGGTG | Primers used in CUT&Tag-qPCR |
| *LpERF1B*-AF | GGAGAGGGAGTTTGAGGATATTGT | Primers used in CUT&Tag-qPCR |
| *LpERF1B*-AR | GAGTGTCTAGCGCGCAATTT | Primers used in CUT&Tag-qPCR |
| *LpERF1B*-BF | GAAGCTCTCTTGACCTCCGA | Primers used in CUT&Tag-qPCR |
| *LpERF1B*-BR | GGATACACAGACGGGCATGA | Primers used in CUT&Tag-qPCR |
| *LpERF1B*-CF | GATCTGTCCAACCTGCAAGC | Primers used in CUT&Tag-qPCR |
| *LpERF1B*-CR | GCGCGGAGAAGGTTAAGATG | Primers used in CUT&Tag-qPCR |
| *LpERF1B*-DF | TTACCATTGTGCATTTGTGAC | Primers used in CUT&Tag-qPCR |
| *LpERF1B*-DR | GTCCATGAGGCCCTTCGATA | Primers used in CUT&Tag-qPCR |
|  |  |  |
| Primers used in cloning and RT-qPCR | **Sequence (5' to 3')** |  |
| *LpCbDR1*-F | ATGTCGATGAGCTTCTTGAGC | LpCbDR1 cloning |
| *LpCbDR1*-R | GAAATGGTTCATCCAATTGGAGC | LpCbDR1 cloning |
| *LpCbDR1*RNAi-F | GCTCTAGAGTCGACAAGGTCGGCTTCTCCGGT | Knockdown target |
| *LpCbDR1*RNAi-R | CCCAAGCTTGAATTCTGTCCTTACCAGTCGCCTTCC | Knockdown target |
| *pLpNOL* | GAAGAAATTAAGCAACGACCCGATC | LpNOL promoter cloning |
| *pLpNOL* | TTGGATTGGTCGTTTGTTC | LpNOL promoter cloning |
| pHis2.1-*pLpNOL-1~-570*-F | CGAGCTCGGTAACCGATGGGGAAG | Y1H assay |
| pHis2.1-*pLpNOL-1~-570*-R | CGACGCGTTCGGGAAGAGCGAGC | Y1H assay |
| pHis2.1-*pLpNOL-571~-1121*-F | CGAGCTCCGTACAAACAAACAACAGTGT | Y1H assay |
| pHis2.1-*pLpNOL-571~-1121*-R | GGACTAGTCATCCTCAAATTACAAAAGGTC | Y1H assay |
| pHis2.1-*pLpNOL-1122~-1672*-F | CGAGCTCTTGGCTAACATAATCCCCTAA | Y1H assay |
| pHis2.1-*pLpNOL-1122~-1672*-R | GGACTAGTTTAATTAATTATTTGATTAGTTTTAACTTAT | Y1H assay |
| pHis2.1-*pLpNOL-1~-1672-*F | CGAGCTCGGTAACCGATGGGGAAG | Y1H assay |
| pHis2.1-*pLpNOL-1~-1672-*R | GGACTAGTTTAATTAATTATTTGATTAGTTTTAACTTAT | Y1H assay |
| *LpCbDR1*-F | GCTCTAGAATGTCGATGAGCTTCTTGAGC | Acitviting activity test |
| *LpCbDR1*-R | CCGGAATTCGAAATGGTTCATCCAATTGGA | Activiting activity test |
| p1305.2-121-F | CCCTGTTGTTTGGTGTTACTT | Transgenic plant test by PCR |
| 730-87-F | GCGTATTTCCGGTCCTTCAGG | Transgenic plant test by PCR |
| *LpCbDR1*-227R | AGAGGACCTCGTAACGCTAG | Transgenic plant test by PCR |
| *LpPLA*-F | ATGCCAACGACGTCGTCG | LpPLA cloning |
| *LpPLA*-R | TTAGGTTTCCTCGAAGTCCCTGA | LpPLA cloning |
| *LpERF1B*-F | ATGGAGCCGCCAAATGATC | LpERF1B cloning |
| *LpERF1B*-R | CAGATCGGACAAGGTGAGC | LpERF1B cloning |
| *LpCbDR1*-F | GCAGGCAGCAATGGCATGAG | qRT-PCR |
| *LpCbDR1*-R | GAGCACCTTCTTGTCACAGTTC | qRT-PCR |
| *LpSGR*-F | GAGGAGGCGAACTCGAAG | qRT-PCR |
| *LpSGR*-R | GGTTGTACCACCCTTGCAG | qRT-PCR |
| *LpPPH*-F | ACCCAGGTGATTCAGGAAAG | qRT-PCR |
| *LpPPH*-R | CCTGACCTCACCAACCTTCT | qRT-PCR |
| *LpPAO*-F | TCAAGGCCAAGAGAAGGTCT | qRT-PCR |
| *LpPAO*-R | TGTGTGGGTGTGAATGTGAG | qRT-PCR |
| *LpNYC1*-F | GATCGTCTCCCAGAAGTGCT | qRT-PCR |
| *LpNYC1*-R | GCCAGTCTCCTGCTTGAAC | qRT-PCR |
| *LpNOL*-F | GCTGGCAAAGAAGTTTCTCA | qRT-PCR |
| *LpNOL*-R | ATGCTGCTCTCCAAATTCCT | qRT-PCR |
| *LpCAO*-F | GATCAAGCACATCCCTTTCA | qRT-PCR |
| *LpCAO*-R | ACAAGCCGAAGATCCTCATT | qRT-PCR |
| *LpPLA*-F | GCGACGACAAATTCCGATCT | qRT-PCR |
| *LpPLA*-R | CGTCTCCGTAGTTCCTCACA | qRT-PCR |
| *LpERF1B*-F | CTGCATCCGGAAGAGAAACC | qRT-PCR |
| *LpERF1B*-R | AGATCGGACAAGGTGAGCAG | qRT-PCR |

**Table S2.** Overlapping genes from the CUT&Tag and RNA-seq.

| NCBI accession number | Mean TPM values of  ‘DHX’ | Mean TPM values of  ‘DHX + Ethanol’ | log2(fc) | PValue | FDR | Symbol | Description |
| --- | --- | --- | --- | --- | --- | --- | --- |
| LOC127323795 | 0.001 | 3.978 | 11.95783 | 2.98E-09 | 5.45E-08 | LpERF1B | XP_037431286.1 ethylene-response factor C3-like [Triticum dicoccoides] |
| LOC127327743 | 0.001 | 0.372333 | 8.540451 | 0.008678 | 0.03966 | LpPLA7 | XP_044347922.1 phospholipase A1-II 7-like [Triticum aestivum] |
| LOC127293342 | 0.606667 | 29.73733 | 5.615227 | 6.28E-68 | 3.43E-65 | Os03g0586800 | XP_037422364.1 lysine--tRNA ligase-like [Triticum dicoccoides] |
| LOC127341796 | 1.184 | 23.93567 | 4.337421 | 9.90E-20 | 4.79E-18 | THRRS | XP_006659275.2 threonine--tRNA ligase, mitochondrial 1 [Oryza brachyantha] |
| LOC127348832 | 0.154333 | 2.000333 | 3.696119 | 1.15E-05 | 0.000117 | WRKY62 | XP_037427938.1 WRKY DNA-binding transcription factor 70-like [Triticum dicoccoides] |
| LOC127295836 | 0.530333 | 5.998333 | 3.49959 | 1.23E-18 | 5.47E-17 | Os03g0291500 | XP_044370169.1 asparagine synthetase [glutamine-hydrolyzing] 1-like [Triticum aestivum] |
| LOC127311849 | 0.106333 | 1.194667 | 3.489942 | 0.001913 | 0.011115 | BC10 | XP_037465652.1 glycosyltransferase BC10-like [Triticum dicoccoides] |
| LOC127330889 | 1.453667 | 14.109 | 3.278847 | 2.67E-08 | 4.25E-07 | ERF1B | VAI00596.1 unnamed protein product [Triticum turgidum subsp. durum] [Triticum turgidum] |
| LOC127322844 | 2.683667 | 23.17733 | 3.110437 | 1.72E-27 | 1.39E-25 | SOT5 | XP_044969653.1 flavonol 3-sulfotransferase-like [Hordeum vulgare subsp. vulgare] [Hordeum vulgare] |
| LOC127327031 | 24.41633 | 183.8127 | 2.912318 | 6.61E-68 | 3.55E-65 | SAM2 | VAI47800.1 unnamed protein product [Triticum turgidum subsp. durum] [Triticum turgidum] |
| LOC127301110 | 3.707333 | 25.65533 | 2.790805 | 4.04E-19 | 1.86E-17 | -- | XP_003571570.1 uncharacterized protein LOC100845602 [Brachypodium distachyon] |
| LOC127314599 | 3.432 | 22.663 | 2.723217 | 5.19E-42 | 9.51E-40 | At2g19130 | KAE8785141.1 putative S-locus receptor-like protein kinase family protein [Hordeum vulgare] |
| LOC127299218 | 71.27033 | 458.5283 | 2.685637 | 3.38E-88 | 4.54E-85 | CYP51 | XP_003573827.1 obtusifoliol 14-alpha demethylase [Brachypodium distachyon] |
| LOC127324010 | 1.083667 | 6.861667 | 2.662638 | 8.17E-18 | 3.42E-16 | CRK6 | XP_037433218.1 putative receptor-like protein kinase At4g00960 [Triticum dicoccoides] |
| LOC127340750 | 0.145 | 0.912 | 2.652981 | 0.007555 | 0.035296 | At1g28600 | XP_044319513.1 GDSL esterase/lipase At1g28600-like [Triticum aestivum] |
| LOC127314111 | 5.861333 | 34.73367 | 2.567034 | 8.38E-40 | 1.39E-37 | At5g44410 | XP_037463487.1 berberine bridge enzyme-like 27 [Triticum dicoccoides] |
| LOC127298131 | 0.168 | 0.983333 | 2.549219 | 0.000593 | 0.004044 | FMO1 | XP_020193809.1 probable flavin-containing monooxygenase 1 isoform X1 [Aegilops tauschii subsp. strangulata] [Aegilops tauschii] |
| LOC127313864 | 0.226 | 1.298 | 2.521896 | 0.001848 | 0.010791 | Os07g0271500 | XP_003561033.1 bisdemethoxycurcumin synthase [Brachypodium distachyon] |
| LOC127314041 | 0.698 | 3.969333 | 2.507598 | 3.31E-06 | 3.76E-05 | -- | XP_037467621.1 uncharacterized protein LOC119339826 [Triticum dicoccoides] |
| LOC127335641 | 1.032 | 5.747333 | 2.47745 | 1.49E-11 | 3.55E-10 | UGT73C6 | XP_037486432.1 UDP-glycosyltransferase 73C6-like [Triticum dicoccoides] |
| LOC127309693 | 1.025 | 4.788 | 2.223799 | 0.000161 | 0.00128 | GDU4 | XP_037446740.1 protein GLUTAMINE DUMPER 4-like [Triticum dicoccoides] |
| LOC127344099 | 53.9 | 251.7057 | 2.223381 | 2.99E-09 | 5.45E-08 | SBT3.5 | VAH80705.1 unnamed protein product [Triticum turgidum subsp. durum] [Triticum turgidum] |
| LOC127336103 | 6.129 | 28.43533 | 2.213961 | 2.14E-42 | 4.01E-40 | B120 | XP_010240474.1 G-type lectin S-receptor-like serine/threonine-protein kinase At1g11330 isoform X1 [Brachypodium distachyon] |
| LOC127313185 | 1.341333 | 6.101333 | 2.185457 | 9.83E-06 | 0.000102 | -- | XP_010227788.1 uncharacterized protein LOC100845166 [Brachypodium distachyon] |
| LOC127301402 | 6.397 | 28.18433 | 2.139426 | 5.42E-35 | 6.88E-33 | RIPK | XP_037434457.1 serine/threonine-protein kinase RIPK-like [Triticum dicoccoides] |
| LOC127316719 | 1.830333 | 7.866 | 2.103524 | 4.26E-09 | 7.63E-08 | NAC090 | XP_044962668.1 NAC domain-containing protein 90-like [Hordeum vulgare subsp. vulgare] [Hordeum vulgare] |
| LOC127334973 | 419.693 | 1683.146 | 2.003754 | 1.96E-57 | 7.53E-55 | OPR11 | XP_040255097.1 putative 12-oxophytodienoate reductase 11 isoform X1 [Aegilops tauschii subsp. strangulata] [Aegilops tauschii] |
| LOC127313172 | 2.562333 | 10.26167 | 2.001735 | 2.81E-11 | 6.47E-10 | Os03g0188200 | XP_020153278.1 E3 ubiquitin-protein ligase Os03g0188200 [Aegilops tauschii subsp. strangulata] [Aegilops tauschii] |
| LOC127307002 | 0.082333 | 0.319333 | 1.955515 | 0.003041 | 0.016528 | ND2 | XP_044343259.1 NADH-ubiquinone oxidoreductase chain 2-like [Triticum aestivum] |
| LOC127296226 | 0.561 | 2.134333 | 1.927713 | 0.000175 | 0.001383 | PK1 | XP_044956253.1 putative receptor protein kinase ZmPK1 [Hordeum vulgare subsp. vulgare] [Hordeum vulgare] |
| LOC127317577 | 0.625 | 2.374333 | 1.925594 | 8.59E-05 | 0.000728 | TCP14 | XP_037468507.1 transcription factor TCP15-like [Triticum dicoccoides] |
| LOC127312953 | 13.56967 | 50.768 | 1.903534 | 5.62E-32 | 5.88E-30 | VQ17 | XP_037456819.1 uncharacterized protein LOC119327844 [Triticum dicoccoides] |
| LOC127305673 | 47.89233 | 175.4593 | 1.87327 | 1.73E-47 | 4.21E-45 | LKR/SDH | XP_044404668.1 alpha-aminoadipic semialdehyde synthase-like isoform X2 [Triticum aestivum] |
| LOC127317802 | 3.275333 | 11.972 | 1.869951 | 2.66E-10 | 5.49E-09 | XIPI | KAF0889603.1 hypothetical protein E2562_029277 [Oryza meyeriana var. granulata] [Oryza meyeriana] |
| LOC127336368 | 16.08967 | 58.78767 | 1.869379 | 2.81E-35 | 3.65E-33 | WAK2 | XP_037479190.1 wall-associated receptor kinase 3-like [Triticum dicoccoides] |
| LOC127319113 | 1.129 | 4.091333 | 1.857526 | 2.26E-05 | 0.000216 | EXPA4 | XP_044977444.1 expansin-A4 [Hordeum vulgare subsp. vulgare] [Hordeum vulgare] |
| LOC127335999 | 6.776 | 24.34133 | 1.844902 | 1.61E-22 | 9.24E-21 | CISZOG1 | XP_020177985.1 cis-zeatin O-glucosyltransferase 1-like [Aegilops tauschii subsp. strangulata] [Aegilops tauschii] |
| LOC127319553 | 2.843667 | 10.15967 | 1.837029 | 1.20E-15 | 4.26E-14 | At2g33490 | XP_020147425.1 uncharacterized protein At2g33490 [Aegilops tauschii subsp. strangulata] [Aegilops tauschii] |
| LOC127317567 | 12.554 | 43.36233 | 1.788295 | 2.28E-29 | 2.00E-27 | NIP2-2 | AOR52535.1 silicon transporter NIP2-2a [Agrostis stolonifera] |
| LOC127335495 | 9.605333 | 32.853 | 1.774118 | 2.63E-24 | 1.74E-22 | SKIP2 | XP_010240100.1 F-box protein At1g47056 [Brachypodium distachyon] |
| LOC127305838 | 5.369667 | 17.16667 | 1.676705 | 1.26E-11 | 3.04E-10 | ARP1 | XP_044953250.1 probable RNA-binding protein ARP1 [Hordeum vulgare subsp. vulgare] [Hordeum vulgare] |
| LOC127323319 | 19.63333 | 61.64633 | 1.65071 | 2.29E-20 | 1.15E-18 | HAK16 | KAE8805340.1 putative potassium transporter 16 [Hordeum vulgare] |
| LOC127296513 | 35.598 | 111.5177 | 1.647404 | 3.25E-37 | 4.66E-35 | sec61a | XP_020146398.1 protein transport protein Sec61 subunit alpha [Aegilops tauschii subsp. strangulata] [Aegilops tauschii] |
| LOC127343667 | 1.276 | 3.98 | 1.64114 | 0.003616 | 0.019103 | -- | XP_003567072.1 uncharacterized protein LOC100834304 [Brachypodium distachyon] |
| LOC127335042 | 0.985 | 2.988 | 1.600985 | 0.000106 | 0.000879 | nep1 | XP_020158555.1 aspartic proteinase nepenthesin-1 [Aegilops tauschii subsp. strangulata] [Aegilops tauschii] |
| LOC127340089 | 3.276667 | 9.931333 | 1.599759 | 5.42E-11 | 1.22E-09 | At5g39980 | XP_020194414.1 pentatricopeptide repeat-containing protein At5g39980, chloroplastic [Aegilops tauschii subsp. strangulata] [Aegilops tauschii] |
| LOC127332371 | 15.27867 | 45.84067 | 1.585109 | 1.08E-18 | 4.83E-17 | FAP3 | XP_010234161.1 fatty-acid-binding protein 3, chloroplastic [Brachypodium distachyon] |
| LOC127302830 | 15.576 | 46.27333 | 1.570856 | 1.30E-34 | 1.61E-32 | FMO1 | XP_020189183.1 probable flavin-containing monooxygenase 1 [Aegilops tauschii subsp. strangulata] [Aegilops tauschii] |
| LOC127310602 | 2.695667 | 7.981333 | 1.565988 | 4.86E-08 | 7.46E-07 | RGA5 | QKY74105.1 powdery mildew resistance protein 12-like [Aegilops speltoides var. ligustica] [Aegilops speltoides] |
| LOC127312993 | 1.199 | 3.548333 | 1.56531 | 0.00059 | 0.004026 | -- | XP_037404767.1 uncharacterized protein LOC119267484 isoform X1 [Triticum dicoccoides] |
| LOC127296170 | 42.806 | 125.999 | 1.557527 | 2.56E-34 | 3.10E-32 | PYL5 | ATP66592.1 PYL5 [Festuca elata] |
| LOC127327634 | 4.811667 | 14.09867 | 1.55095 | 4.03E-10 | 8.10E-09 | OsI_21081 | XP_044405797.1 phospholipase A1-II 7-like [Triticum aestivum] |
| LOC127323311 | 11.036 | 32.07033 | 1.539022 | 1.55E-18 | 6.85E-17 | At2g27500 | KAF6990374.1 hypothetical protein CFC21_007575 [Triticum aestivum] |
| LOC127311218 | 0.699333 | 2.025 | 1.53387 | 3.75E-05 | 0.000343 | At3g47110 | VAI77746.1 unnamed protein product [Triticum turgidum subsp. durum] [Triticum turgidum] |
| LOC127336881 | 41.803 | 119.4043 | 1.514177 | 2.99E-23 | 1.82E-21 | PGDH1 | XP_003580703.1 D-3-phosphoglycerate dehydrogenase 1, chloroplastic [Brachypodium distachyon] |
| LOC127307427 | 7.978 | 22.61533 | 1.503202 | 2.80E-19 | 1.31E-17 | AZG1 | XP_024314670.1 adenine/guanine permease AZG1 [Brachypodium distachyon] |
| LOC127304797 | 1.879667 | 5.311333 | 1.498597 | 0.001131 | 0.007078 | -- | XP_020163786.1 uncharacterized protein LOC109749220 [Aegilops tauschii subsp. strangulata] [Aegilops tauschii] |
| LOC127336623 | 18.956 | 52.74833 | 1.476471 | 1.33E-17 | 5.53E-16 | AAE3 | XP_044968632.1 oxalate--CoA ligase [Hordeum vulgare subsp. vulgare] [Hordeum vulgare] |
| LOC127314595 | 16.304 | 44.76467 | 1.457134 | 6.20E-24 | 3.97E-22 | CRPK1 | XP_010234443.1 cold-responsive protein kinase 1 isoform X4 [Brachypodium distachyon] |
| LOC127326428 | 1.902333 | 5.179333 | 1.444996 | 1.47E-06 | 1.78E-05 | MST1 | XP_020180548.1 sugar transport protein MST1 [Aegilops tauschii subsp. strangulata] [Aegilops tauschii] |
| LOC127312119 | 32.14167 | 86.65133 | 1.430777 | 2.91E-29 | 2.54E-27 | CRPK1 | XP_037459714.1 cold-responsive protein kinase 1-like [Triticum dicoccoides] |
| LOC127316550 | 75.79933 | 204.1037 | 1.429045 | 4.09E-31 | 4.07E-29 | BANGLUC | CAA78834.1 (1-3, 1-4)-beta-glucanase [Avena sativa] |
| LOC127341560 | 3.926333 | 10.502 | 1.41941 | 4.33E-08 | 6.68E-07 | WRKY72 | XP_020189817.1 probable WRKY transcription factor 72 [Aegilops tauschii subsp. strangulata] [Aegilops tauschii] |
| LOC127314052 | 5.282333 | 14.06567 | 1.412931 | 8.23E-06 | 8.69E-05 | -- | XP_037440008.1 uncharacterized protein LOC119307994 [Triticum dicoccoides] |
| LOC127292607 | 1.968 | 5.236 | 1.411735 | 4.23E-05 | 0.000383 | -- | XP_037431483.1 uncharacterized protein LOC119298048 [Triticum dicoccoides] |
| LOC127296040 | 1.913667 | 5.031 | 1.394506 | 0.000154 | 0.001228 | AHL22 | XP_020200949.1 AT-hook motif nuclear-localized protein 29 [Aegilops tauschii subsp. strangulata] [Aegilops tauschii] |
| LOC127336495 | 14.88933 | 39.07967 | 1.392139 | 2.75E-23 | 1.68E-21 | NPF5.10 | XP_044327107.1 protein NRT1/ PTR FAMILY 5.10-like [Triticum aestivum] |
| LOC127292688 | 266.3893 | 692.8733 | 1.379055 | 2.38E-34 | 2.90E-32 | MEE14 | XP_003558937.1 CCG-binding protein 1 [Brachypodium distachyon] |
| LOC127317005 | 83.02867 | 212.7253 | 1.35731 | 1.62E-24 | 1.11E-22 | CYP76B6 | XP_020157227.1 geraniol 8-hydroxylase [Aegilops tauschii subsp. strangulata] [Aegilops tauschii] |
| LOC127314280 | 5.842333 | 14.92833 | 1.353437 | 1.49E-13 | 4.34E-12 | -- | XP_044408235.1 uncharacterized protein LOC123132496 isoform X1 [Triticum aestivum] |
| LOC127344788 | 24.83667 | 63.36967 | 1.351321 | 1.76E-25 | 1.28E-23 | PBL15 | XP_044341900.1 probable serine/threonine-protein kinase PBL1 [Triticum aestivum] |
| LOC127308782 | 3.693333 | 9.288667 | 1.330548 | 2.63E-15 | 8.95E-14 | RGA4 | XP_037451947.1 putative disease resistance protein RGA3 [Triticum dicoccoides] |
| LOC127346728 | 2.175333 | 5.424 | 1.318121 | 1.57E-05 | 0.000157 | AT4 | XP_044448320.1 acyl transferase 4-like [Triticum aestivum] |
| LOC127341625 | 107.2073 | 266.3573 | 1.312959 | 1.34E-26 | 1.04E-24 | GI | ABF83898.2 GIGANTEA [Lolium perenne] |
| LOC127294545 | 9.245 | 22.96067 | 1.312419 | 5.31E-13 | 1.48E-11 | CIPK14 | AEZ51505.1 CBL-interacting protein kinase 14 [Hordeum vulgare subsp. spontaneum] [Hordeum vulgare] |
| LOC127335607 | 6.665 | 16.38267 | 1.297493 | 1.56E-08 | 2.57E-07 | Dctpp1 | XP_044970331.1 dCTP pyrophosphatase 1-like [Hordeum vulgare subsp. vulgare] [Hordeum vulgare] |
| LOC127334343 | 1.766 | 4.311667 | 1.28776 | 0.000824 | 0.005379 | -- | XP_044324224.1 uncharacterized protein LOC123045287 [Triticum aestivum] |
| LOC127317179 | 5.217667 | 12.623 | 1.274578 | 1.36E-12 | 3.62E-11 | -- | XP_014753209.1 uncharacterized protein LOC100823329 [Brachypodium distachyon] |
| LOC127302122 | 6.609333 | 15.95133 | 1.2711 | 9.31E-11 | 2.04E-09 | PILS1 | XP_010238302.1 protein PIN-LIKES 3 [Brachypodium distachyon] |
| LOC127345247 | 222.0503 | 532.9573 | 1.263133 | 1.48E-25 | 1.08E-23 | GSH1-1 | XP_003569058.1 glutamate--cysteine ligase B, chloroplastic [Brachypodium distachyon] |
| LOC127301493 | 4.851333 | 11.601 | 1.257796 | 1.43E-14 | 4.56E-13 | GLR2.9 | XP_044378780.1 glutamate receptor 2.9-like [Triticum aestivum] |
| LOC127327456 | 0.596 | 1.423 | 1.255551 | 0.011055 | 0.048259 | LRK10L-1.2 | XP_044451566.1 LEAF RUST 10 DISEASE-RESISTANCE LOCUS RECEPTOR-LIKE PROTEIN KINASE-like 1.2 isoform X1 [Triticum aestivum] |
| LOC127333224 | 0.919667 | 2.158333 | 1.230735 | 0.003305 | 0.017705 | CRK6 | XP_020155785.1 cysteine-rich receptor-like protein kinase 6 [Aegilops tauschii subsp. strangulata] [Aegilops tauschii] |
| LOC127293715 | 22.06867 | 51.39833 | 1.219722 | 1.16E-17 | 4.85E-16 | CAT1 | XP_044984637.1 cationic amino acid transporter 1 [Hordeum vulgare subsp. vulgare] [Hordeum vulgare] |
| LOC127344090 | 23.816 | 55.24867 | 1.214009 | 5.02E-14 | 1.53E-12 | -- | XP_020164095.1 uncharacterized protein LOC109749566 [Aegilops tauschii subsp. strangulata] [Aegilops tauschii] |
| LOC127343233 | 18.561 | 43.00767 | 1.212319 | 5.08E-20 | 2.48E-18 | MKP1 | XP_037448824.1 protein-tyrosine-phosphatase MKP1-like isoform X1 [Triticum dicoccoides] |
| LOC127320975 | 6.727667 | 15.552 | 1.208922 | 7.30E-11 | 1.62E-09 | CAMRLK | XP_003571158.2 calmodulin-binding receptor kinase CaMRLK [Brachypodium distachyon] |
| LOC127305635 | 0.966667 | 2.180667 | 1.173679 | 0.000615 | 0.004171 | SMAX1 | XP_037455436.1 protein SMAX1-like [Triticum dicoccoides] |
| LOC127295902 | 119.9083 | 269.755 | 1.169718 | 2.21E-19 | 1.04E-17 | GSTZ5 | VAH89598.1 unnamed protein product [Triticum turgidum subsp. durum] [Triticum turgidum] |
| LOC127302049 | 9.290333 | 20.89367 | 1.169263 | 2.57E-16 | 9.76E-15 | At1g59620 | KAE8777358.1 Disease resistance RPP8-like protein 3 [Hordeum vulgare] |
| LOC127296901 | 19.862 | 44.04433 | 1.148946 | 9.32E-15 | 3.02E-13 | CDF1 | QTZ25447.1 CDF1 [Lolium arundinaceum] |
| LOC127314152 | 9.206 | 20.18867 | 1.132899 | 5.29E-10 | 1.05E-08 | At5g39450 | VAI76523.1 unnamed protein product [Triticum turgidum subsp. durum] [Triticum turgidum] |
| LOC127333988 | 1.003333 | 2.196667 | 1.130515 | 0.000371 | 0.002689 | SIT2 | XP_025810368.1 L-type lectin-domain containing receptor kinase IV.1-like [Panicum hallii] |
| LOC127333140 | 35.414 | 75.98833 | 1.101458 | 7.27E-21 | 3.74E-19 | At2g19130 | BAK05829.1 predicted protein [Hordeum vulgare subsp. vulgare] [Hordeum vulgare] |
| LOC127316342 | 25.15467 | 53.23633 | 1.081585 | 1.85E-15 | 6.40E-14 | CYP71Z18 | XP_044441834.1 zealexin A1 synthase-like [Triticum aestivum] |
| LOC127299686 | 9.908333 | 20.849 | 1.073264 | 1.20E-10 | 2.59E-09 | FUT13 | XP_037440117.1 alpha-(1,4)-fucosyltransferase-like isoform X2 [Triticum dicoccoides] |
| LOC127328270 | 0.936 | 1.964333 | 1.069459 | 0.000516 | 0.003592 | At1g49360 | VAH41910.1 unnamed protein product [Triticum turgidum subsp. durum] [Triticum turgidum] |
| LOC127293867 | 10.876 | 22.81633 | 1.068919 | 9.72E-07 | 1.21E-05 | -- | XP_003566374.1 uncharacterized protein LOC100831532 [Brachypodium distachyon] |
| LOC127299097 | 2.187 | 4.581667 | 1.066919 | 4.47E-06 | 4.97E-05 | At3g47110 | XP_003579254.1 probable LRR receptor-like serine/threonine-protein kinase At3g47570 [Brachypodium distachyon] |
| LOC127334015 | 38.82833 | 81.311 | 1.066341 | 5.97E-14 | 1.81E-12 | -- | XP_044959217.1 chitinase 1-like [Hordeum vulgare subsp. vulgare] [Hordeum vulgare] |
| LOC127296409 | 2.594667 | 5.308667 | 1.0328 | 0.000214 | 0.001649 | ACR8 | VAH88823.1 unnamed protein product [Triticum turgidum subsp. durum] [Triticum turgidum] |
| LOC127332564 | 2.122333 | 4.338667 | 1.0316 | 0.007061 | 0.033373 | -- | RLN19051.1 zinc finger CCCH domain-containing protein 18-like [Panicum miliaceum] |
| LOC127342448 | 2.351333 | 4.778333 | 1.023028 | 0.000257 | 0.001936 | agdD | XP_044348514.1 sulfoquinovosidase-like [Triticum aestivum] |
| LOC127318560 | 37.101 | 74.756 | 1.010731 | 1.40E-11 | 3.35E-10 | ACA7 | XP_037423309.1 alpha carbonic anhydrase 7-like [Triticum dicoccoides] |
| LOC127305936 | 5.024 | 10.11867 | 1.010111 | 7.54E-05 | 0.000645 | -- | XP_044414369.1 uncharacterized protein LOC123138455 [Triticum aestivum] |
| LOC127314881 | 150.494 | 301.8977 | 1.004354 | 8.95E-19 | 4.02E-17 | OMT2 | AAK68907.1 caffeic acid O-methyltransferase [Lolium arundinaceum] |
| LOC127321965 | 18.80633 | 9.308 | -1.01468 | 4.13E-09 | 7.41E-08 | LHA1 | XP_010228254.1 plasma membrane ATPase 3 isoform X2 [Brachypodium distachyon] |
| LOC127305355 | 61.70233 | 30.35933 | -1.02318 | 7.52E-10 | 1.47E-08 | sll0005 | BAJ90114.1 predicted protein [Hordeum vulgare subsp. vulgare] [Hordeum vulgare] |
| LOC127293090 | 67.17 | 32.80467 | -1.03392 | 2.07E-08 | 3.35E-07 | 5MAT | VAH94883.1 unnamed protein product [Triticum turgidum subsp. durum] [Triticum turgidum] |
| LOC127317366 | 482.121 | 233.5417 | -1.04572 | 2.19E-18 | 9.57E-17 | NUDT21 | XP_044958766.1 nudix hydrolase 21, chloroplastic-like [Hordeum vulgare subsp. vulgare] [Hordeum vulgare] |
| LOC127304169 | 3.213 | 1.552 | -1.04979 | 0.006819 | 0.032394 | -- | RLM98118.1 putative transposase [Panicum miliaceum] |
| LOC127309030 | 6.889 | 3.325667 | -1.05065 | 0.000259 | 0.001949 | KAN2 | XP_044953864.1 probable transcription factor KAN2 isoform X1 [Hordeum vulgare subsp. vulgare] [Hordeum vulgare] |
| LOC127327354 | 1069.217 | 510.369 | -1.06694 | 1.32E-14 | 4.22E-13 | At1g56190 | XP_003568189.2 phosphoglycerate kinase, chloroplastic [Brachypodium distachyon] |
| LOC127316854 | 26.24467 | 12.462 | -1.07449 | 1.90E-10 | 3.99E-09 | BHLH130 | VAI73941.1 unnamed protein product [Triticum turgidum subsp. durum] [Triticum turgidum] |
| LOC127296360 | 177.7493 | 83.41933 | -1.09139 | 6.21E-17 | 2.45E-15 | -- | XP_037425627.1 neurofilament heavy polypeptide-like [Triticum dicoccoides] |
| LOC127305963 | 12.15567 | 5.634 | -1.1094 | 0.000826 | 0.005391 | ARG7 | PUZ77818.1 hypothetical protein GQ55_1G403700 [Panicum hallii var. hallii] [Panicum hallii] |
| LOC127318415 | 177.1647 | 81.79933 | -1.11493 | 2.27E-20 | 1.14E-18 | GAD | XP_003574600.1 glutamate decarboxylase [Brachypodium distachyon] |
| LOC127332192 | 4.132667 | 1.851667 | -1.15825 | 0.000263 | 0.001975 | -- | KAF0913585.1 hypothetical protein E2562_023299 [Oryza meyeriana var. granulata] [Oryza meyeriana] |
| LOC127336147 | 245.1717 | 107.8463 | -1.18482 | 9.64E-23 | 5.70E-21 | CRPK1 | XP_037487234.1 cold-responsive protein kinase 1-like isoform X1 [Triticum dicoccoides] |
| LOC127334268 | 73.44133 | 32.219 | -1.18868 | 3.75E-21 | 1.99E-19 | EXO70H1 | XP_044324167.1 exocyst complex component EXO70H1-like [Triticum aestivum] |
| LOC127341853 | 2.121333 | 0.928333 | -1.19226 | 0.008019 | 0.037139 | At4g23740 | XP_044356081.1 probable inactive receptor kinase At4g23740 [Triticum aestivum] |
| LOC127335877 | 35.14733 | 15.064 | -1.22231 | 2.28E-15 | 7.80E-14 | DTX49 | XP_044325506.1 protein DETOXIFICATION 49-like [Triticum aestivum] |
| LOC127336784 | 6.779667 | 2.883667 | -1.23331 | 0.000471 | 0.003316 | FLS | XP_044952619.1 flavonol synthase/flavanone 3-hydroxylase [Hordeum vulgare subsp. vulgare] [Hordeum vulgare] |
| LOC127306870 | 102.614 | 43.327 | -1.24389 | 6.00E-25 | 4.20E-23 | At4g16820 | XP_010235782.2 phospholipase A1-Ibeta2, chloroplastic [Brachypodium distachyon] |
| LOC127292989 | 113.0427 | 47.61033 | -1.24752 | 5.32E-11 | 1.20E-09 | -- | VAI02170.1 unnamed protein product [Triticum turgidum subsp. durum] [Triticum turgidum] |
| LOC127307658 | 24.72367 | 10.35667 | -1.25533 | 1.73E-09 | 3.25E-08 | UGT75L6 | AZQ26925.1 UGT75J3 [Avena strigosa] |
| LOC127295653 | 99.612 | 41.39067 | -1.26701 | 9.20E-17 | 3.62E-15 | AAE7 | XP_037430636.1 acetate/butyrate--CoA ligase AAE7, peroxisomal-like [Triticum dicoccoides] |
| LOC127345083 | 65.127 | 26.54433 | -1.29485 | 4.97E-18 | 2.10E-16 | BT3 | XP_010232680.1 BTB/POZ and TAZ domain-containing protein 3 isoform X1 [Brachypodium distachyon] |
| LOC127298466 | 21.19233 | 8.575 | -1.30533 | 8.46E-09 | 1.45E-07 | ZAT6 | QDA34127.1 ZFP2 [Thinopyrum elongatum] |
| LOC127323931 | 106.435 | 42.41167 | -1.32744 | 1.02E-27 | 8.36E-26 | PUMP5 | XP_037442662.1 mitochondrial uncoupling protein 5-like [Triticum dicoccoides] |
| LOC127346209 | 30.73633 | 11.748 | -1.38753 | 2.29E-11 | 5.34E-10 | DI19-5 | XP_003565167.1 protein DEHYDRATION-INDUCED 19 homolog 5 isoform X2 [Brachypodium distachyon] |
| LOC127313955 | 189.0743 | 71.26333 | -1.40772 | 1.88E-19 | 8.93E-18 | PEPC | XP_020163873.1 phosphoenolpyruvate carboxylase 1 [Aegilops tauschii subsp. strangulata] [Aegilops tauschii] |
| LOC127342746 | 272.607 | 99.42133 | -1.4552 | 3.89E-38 | 5.81E-36 | -- | XP_044348930.1 protein ANTAGONIST OF LIKE HETEROCHROMATIN PROTEIN 1-like [Triticum aestivum] |
| LOC127326928 | 48.725 | 17.52433 | -1.4753 | 1.21E-29 | 1.08E-27 | TPS1 | XP_003566035.1 alpha,alpha-trehalose-phosphate synthase [UDP-forming] 1 [Brachypodium distachyon] |
| LOC127305812 | 79.24567 | 28.12033 | -1.49472 | 5.56E-21 | 2.90E-19 | CYP89A2 | XP_044971428.1 cytochrome P450 89A2-like [Hordeum vulgare subsp. vulgare] [Hordeum vulgare] |
| LOC127344457 | 116.5343 | 41.23033 | -1.49898 | 1.62E-32 | 1.76E-30 | P5CS2 | AGQ04179.1 putative delta-1-pyrroline-5-carboxylate 1 [Lolium perenne] |
| LOC127300154 | 2.515667 | 0.866333 | -1.53795 | 8.78E-05 | 0.00074 | -- | XP_044423029.1 uncharacterized protein LOC123147784 isoform X1 [Triticum aestivum] |
| LOC127292627 | 5.131333 | 1.726667 | -1.57134 | 4.08E-09 | 7.33E-08 | BAM1 | XP_003558681.1 leucine-rich repeat receptor-like serine/threonine-protein kinase BAM1 [Brachypodium distachyon] |
| LOC127336887 | 84.737 | 28.407 | -1.57675 | 1.42E-28 | 1.19E-26 | Os04g0650300 | BAJ87418.1 predicted protein [Hordeum vulgare subsp. vulgare] [Hordeum vulgare] |
| LOC127296766 | 9.924667 | 2.974 | -1.73861 | 1.09E-08 | 1.85E-07 | MO2 | XP_044362389.1 monooxygenase 2-like [Triticum aestivum] |
| LOC127301362 | 655.8883 | 195.5043 | -1.74625 | 7.03E-41 | 1.21E-38 | TIFY11B | XP_037426352.1 protein TIFY 11b-like [Triticum dicoccoides] |
| LOC127306099 | 4.213 | 1.244 | -1.75986 | 0.000298 | 0.002211 | At5g39570 | XP_044950626.1 uncharacterized protein At5g39570-like [Hordeum vulgare subsp. vulgare] [Hordeum vulgare] |
| LOC127335996 | 17.73467 | 4.985667 | -1.83071 | 1.64E-15 | 5.72E-14 | SB20O07.14 | BAJ94231.1 predicted protein [Hordeum vulgare subsp. vulgare] [Hordeum vulgare] |
| LOC127299647 | 765.0317 | 202.0953 | -1.92048 | 1.58E-70 | 9.97E-68 | AAP3 | XP_044948509.1 amino acid permease 3-like [Hordeum vulgare subsp. vulgare] [Hordeum vulgare] |
| LOC127327570 | 877.6243 | 212.5123 | -2.04606 | 1.49E-74 | 1.09E-71 | GRXS5 | XP_037473476.1 monothiol glutaredoxin-S9-like [Triticum dicoccoides] |
| LOC127335821 | 159.1003 | 34.727 | -2.19581 | 7.35E-49 | 1.93E-46 | ERF1A | XP_044458682.1 ethylene-responsive transcription factor 2-like [Triticum aestivum] |
| LOC127321948 | 3.416667 | 0.706667 | -2.27349 | 0.001762 | 0.010374 | -- | - |
| LOC127306027 | 123.3053 | 25.482 | -2.27468 | 7.03E-48 | 1.74E-45 | -- | XP_044416858.1 uncharacterized protein LOC123141857 [Triticum aestivum] |
| LOC127309975 | 2.148 | 0.441 | -2.28414 | 0.000385 | 0.002778 | RE2 | XP_037419334.1 probable LRR receptor-like serine/threonine-protein kinase At3g47570 [Triticum dicoccoides] |
| LOC127302740 | 3.206667 | 0.647 | -2.30924 | 1.04E-11 | 2.53E-10 | MIK2 | VAI20572.1 unnamed protein product [Triticum turgidum subsp. durum] [Triticum turgidum] |
| LOC127341129 | 1.426333 | 0.277667 | -2.36089 | 0.002126 | 0.012184 | GAT1_2.1 | XP_037417358.1 putative glutamine amidotransferase GAT1_2.1 [Triticum dicoccoides] |
| LOC127343494 | 1.263667 | 0.240667 | -2.39251 | 0.004894 | 0.024543 | MAPKKK17 | XP_037409844.1 mitogen-activated protein kinase kinase kinase 17-like [Triticum dicoccoides] |
| LOC127301696 | 0.502333 | 0.085333 | -2.55746 | 0.011242 | 0.048919 | CSLA11 | XP_003572230.2 probable mannan synthase 11 [Brachypodium distachyon] |
| LOC127335396 | 0.583 | 0.086333 | -2.75551 | 0.003929 | 0.020458 | WAK2 | VAI68456.1 unnamed protein product [Triticum turgidum subsp. durum] [Triticum turgidum] |
| LOC127309966 | 2.632 | 0.368667 | -2.83577 | 0.003242 | 0.017439 | ycf3 | AJN90436.1 photosystem I assembly protein ycf3 [Phyllostachys edulis] |
| LOC127300162 | 5.273333 | 0.556667 | -3.24383 | 4.93E-06 | 5.43E-05 | CML19 | XP_044948109.1 probable calcium-binding protein CML25/26 [Hordeum vulgare subsp. vulgare] [Hordeum vulgare] |
| LOC127326257 | 1.185667 | 0.018667 | -5.98909 | 7.73E-06 | 8.22E-05 | co-2 | XP_020150179.1 polyphenol oxidase I, chloroplastic [Aegilops tauschii subsp. strangulata] [Aegilops tauschii] |

**Table S3.** Predicated DNA motifs bound by LpCbDR1 according to CUT&Tag

| Rank | Motif | P-value | % of Targets | % of Background |
| --- | --- | --- | --- | --- |
| 1 | 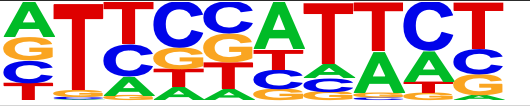 | 1e-23 | 35.25% | 25.73% |
| 2 | 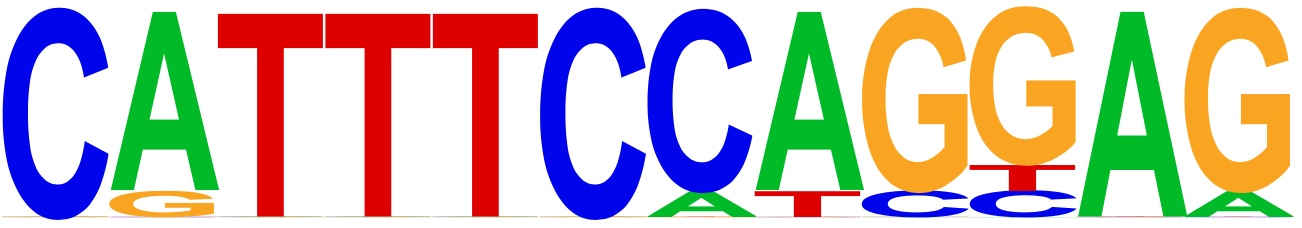 | 1e-22 | 0.48% | 0.00% |
| 3 | 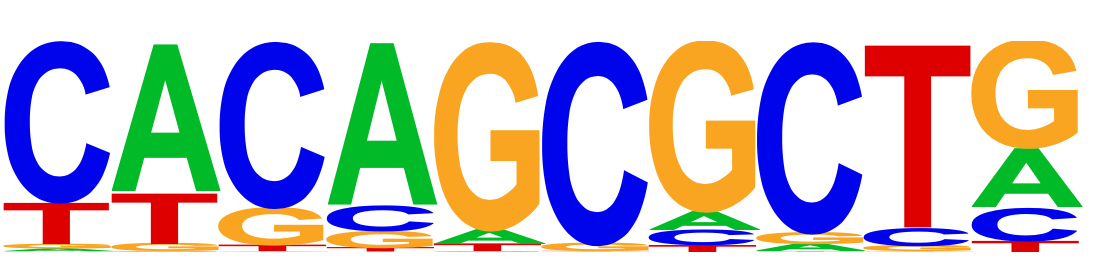 | 1e-20 | 4.13% | 1.32% |
| 4 | 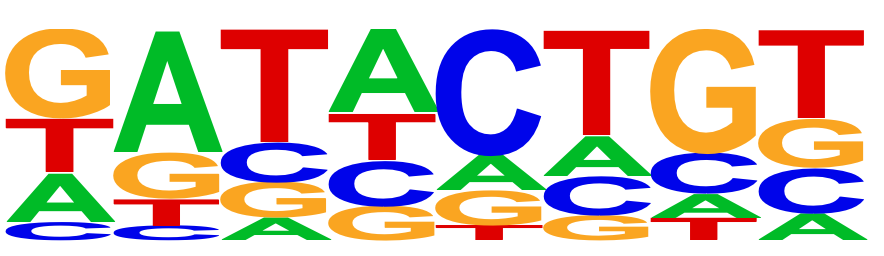 | 1e-19 | 29.76% | 21.62% |
| 5 | 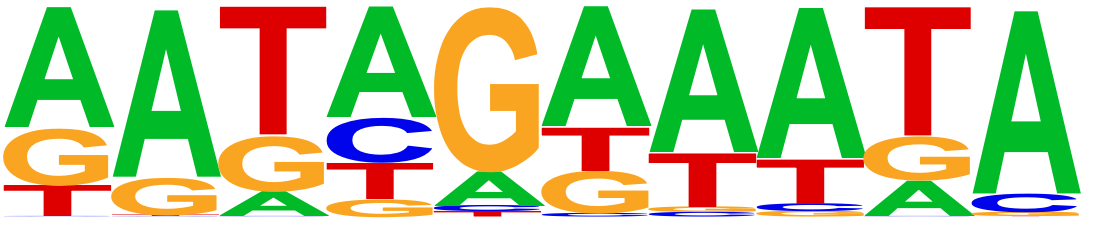 | 1e-17 | 5.14% | 2.05% |
| 6 | 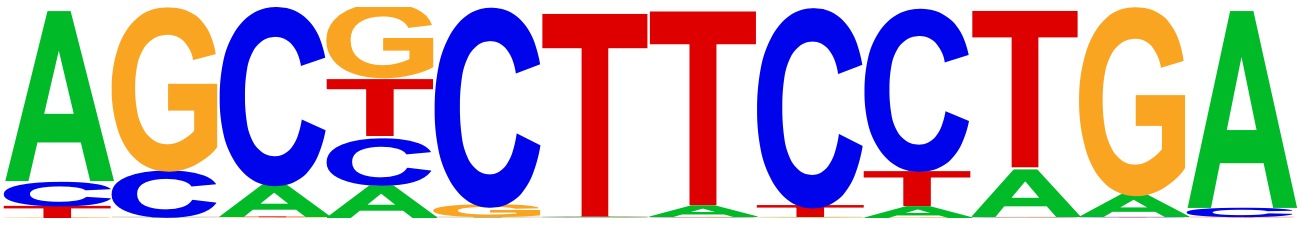 | 1e-16 | 1.14% | 0.12% |
| 7 | 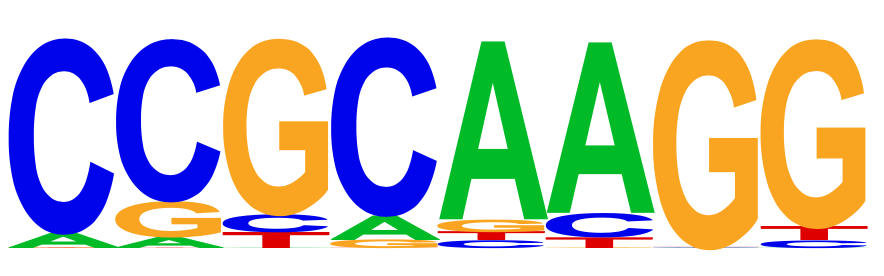 | 1e-15 | 10.32% | 5.86% |
| 8 | 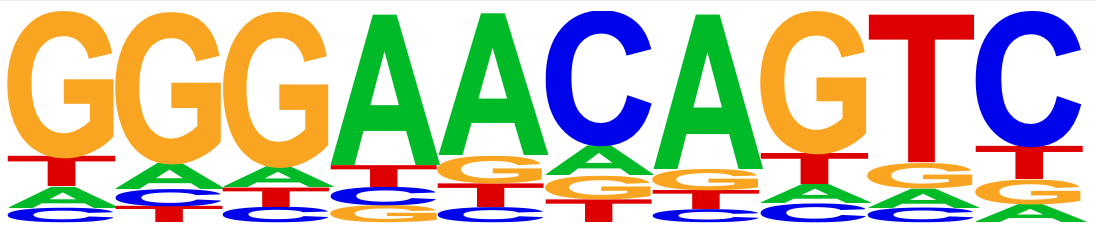 | 1e-15 | 26.03% | 18.99% |
| 9 | 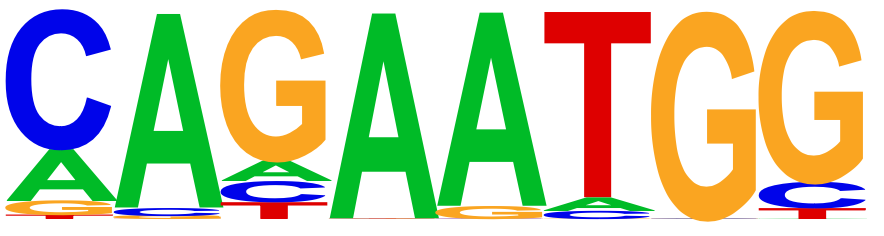 | 1e-15 | 9.88% | 5.54% |
| 10 | 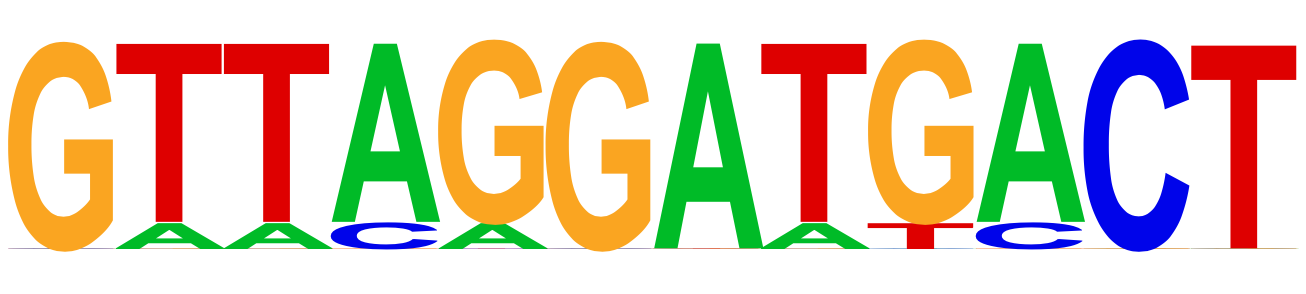 | 1e-15 | 45.17% | 36.84% |
| 11 | 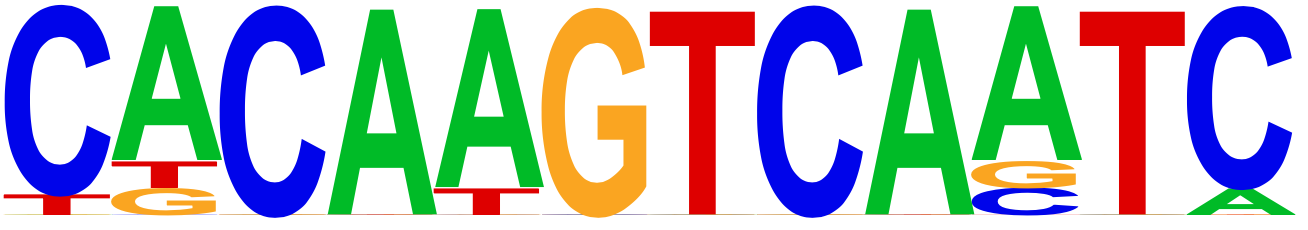 | 1e-15 | 0.35% | 0.00% |
| 12 | 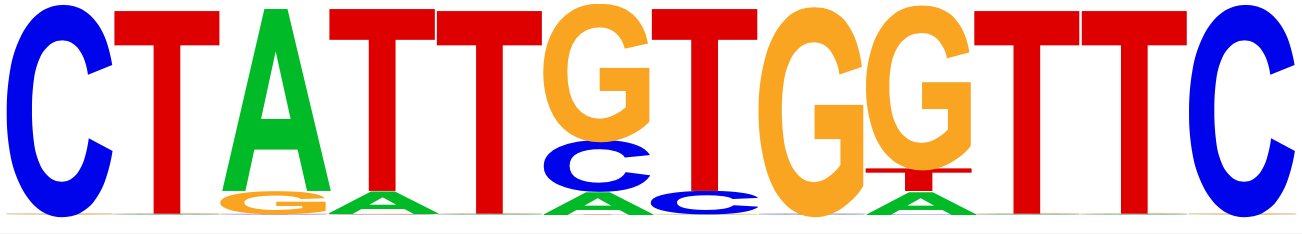 | 1e-13 | 0.44% | 0.01% |
| 13 | 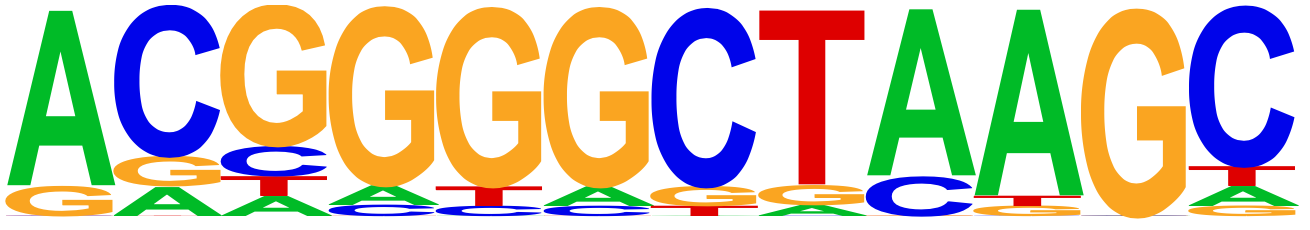 | 1e-13 | 0.92% | 0.10% |
| 14 | 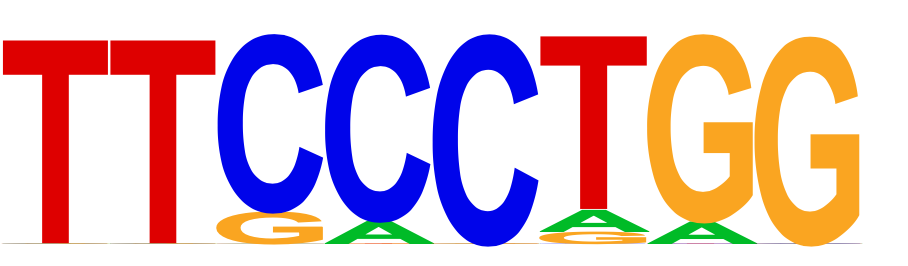 | 1e-13 | 59.75% | 51.94% |
| 15 | 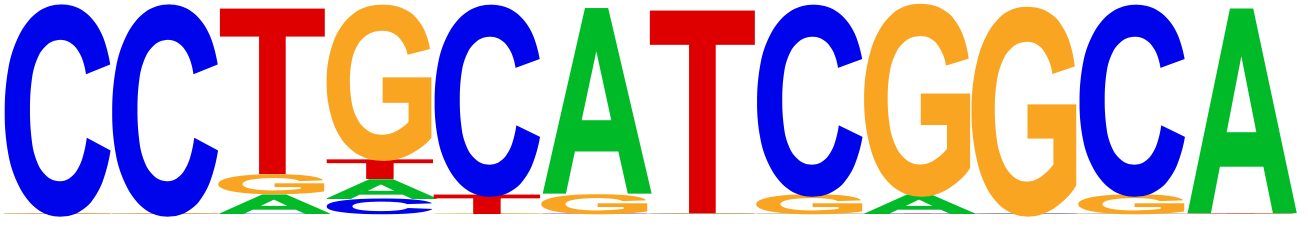 | 1e-12 | 0.48% | 0.02% |
| 16 | 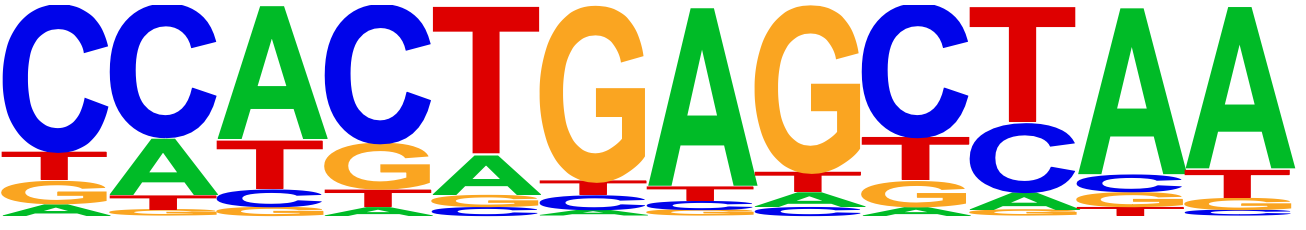 | 1e-12 | 0.31% | 0.00% |
| 17 | 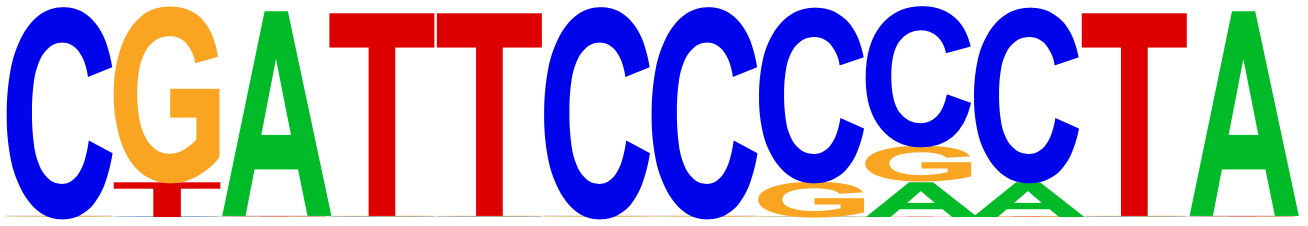 | 1e-12 | 0.31% | 0.00% |
| 18 | 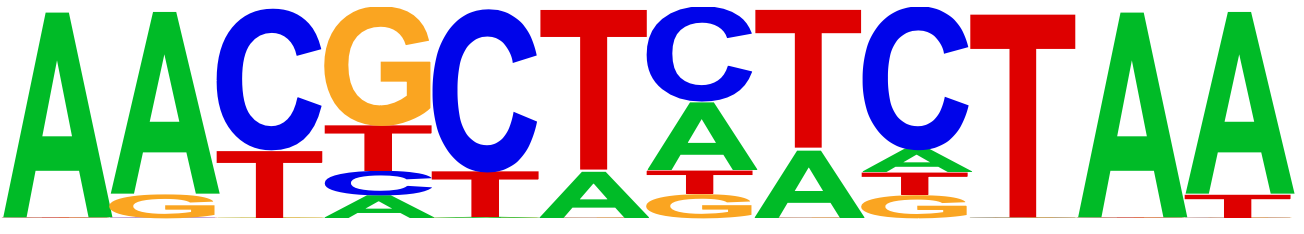 | 1e-12 | 0.70% | 0.05% |
| 19 | 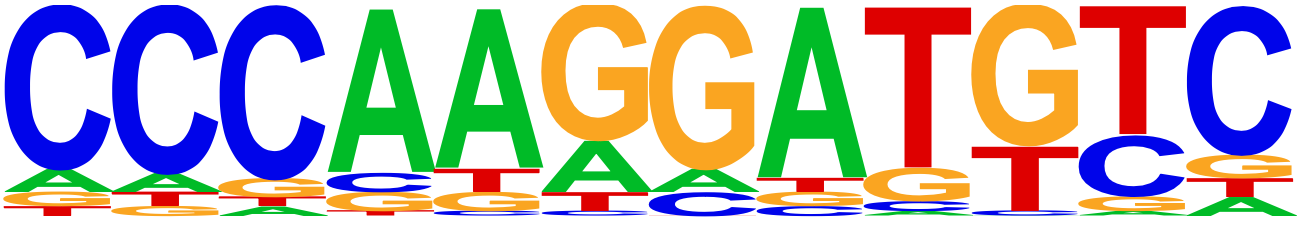 | 1e-12 | 0.35% | 0.01% |
| 20 | 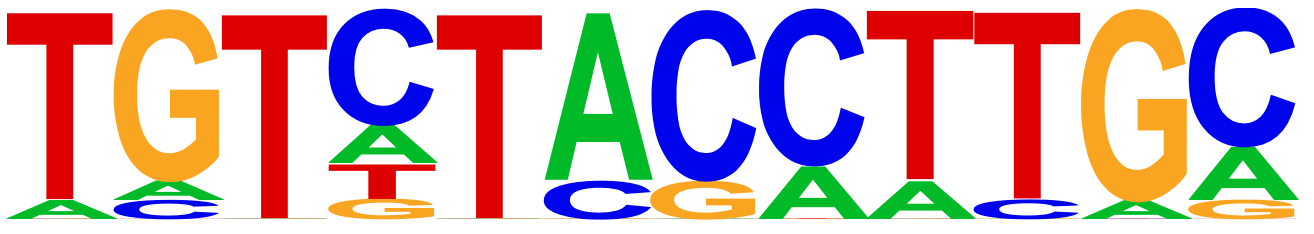 | 1e-12 | 1.01% | 0.13% |
| 21 | 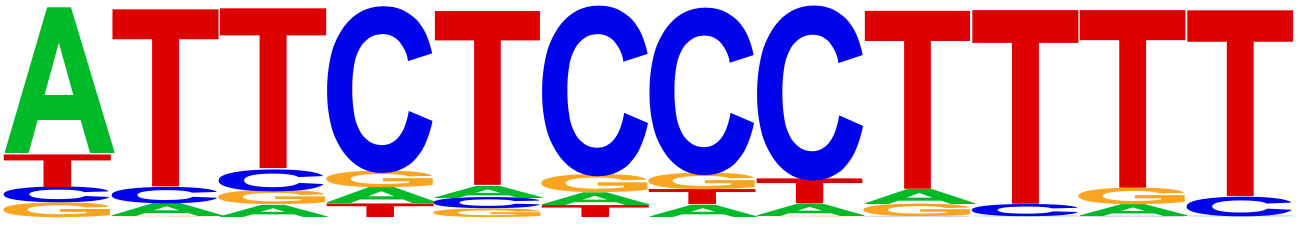 | 1e-12 | 3.64% | 1.49% |
| 22 | 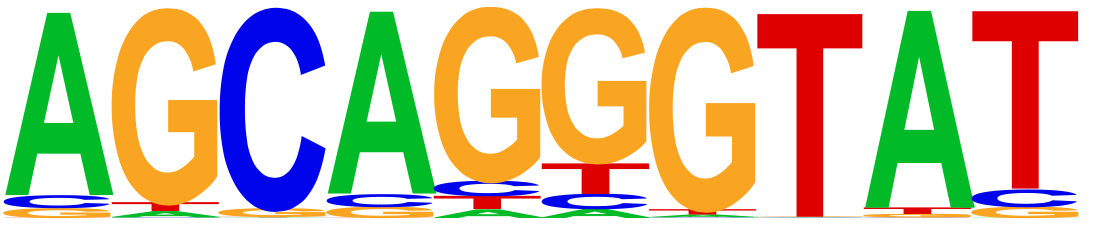 | 1e-12 | 1.62% | 0.38% |
| 23 * | 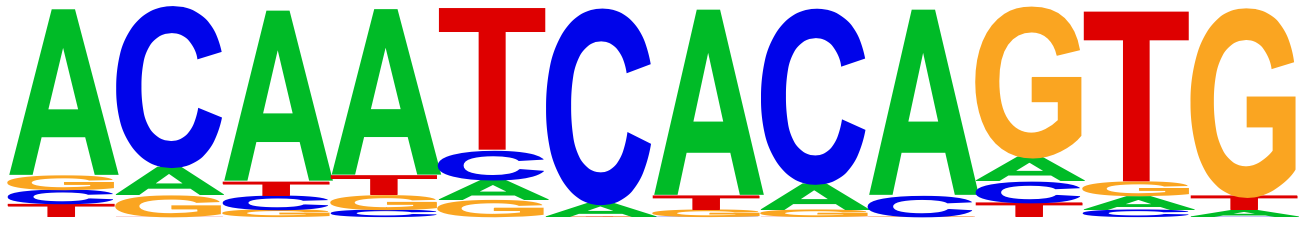 | 1e-11 | 2.15% | 0.65% |
| 24 * | 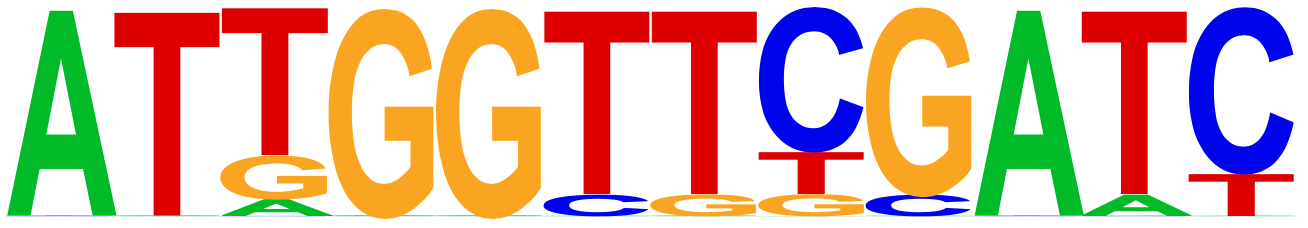 | 1e-11 | 0.57% | 0.03% |
| 25 * | 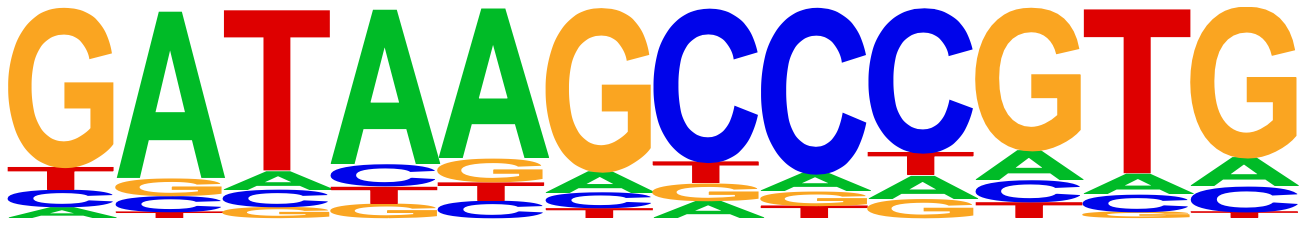 | 1e-11 | 56.80% | 49.62% |
| 26 * | 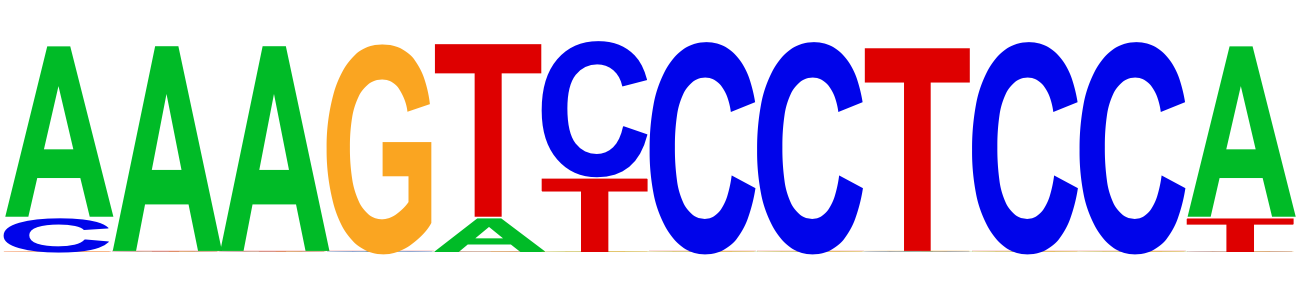 | 1e-11 | 0.35% | 0.01% |
| 27 * | 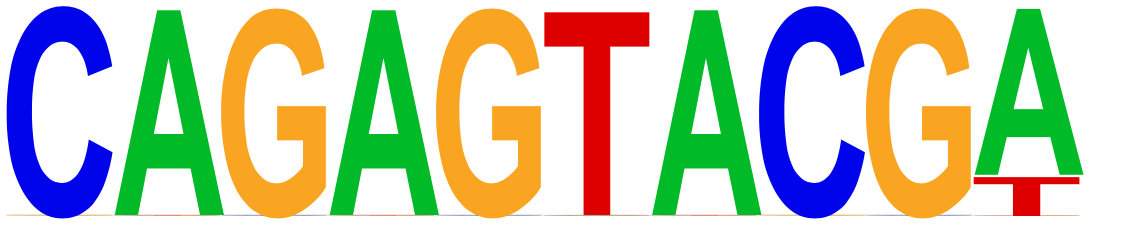 | 1e-11 | 66.29% | 59.32% |
| 28 * | 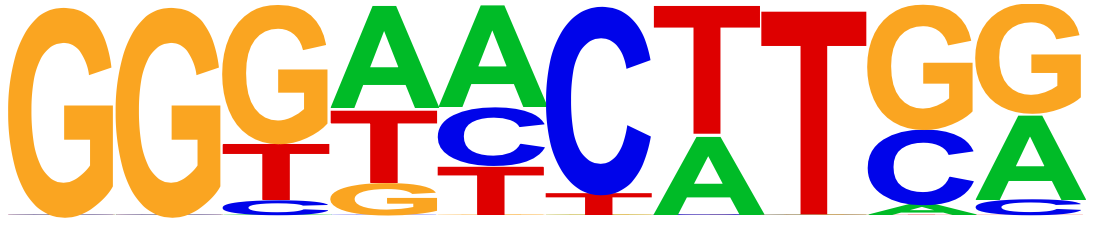 | 1e-11 | 2.99% | 1.16% |
| 29 * | 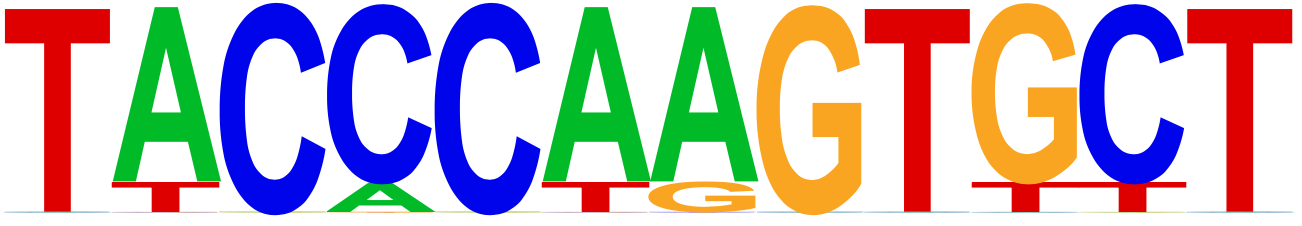 | 1e-10 | 0.31% | 0.01% |
| 30 * | 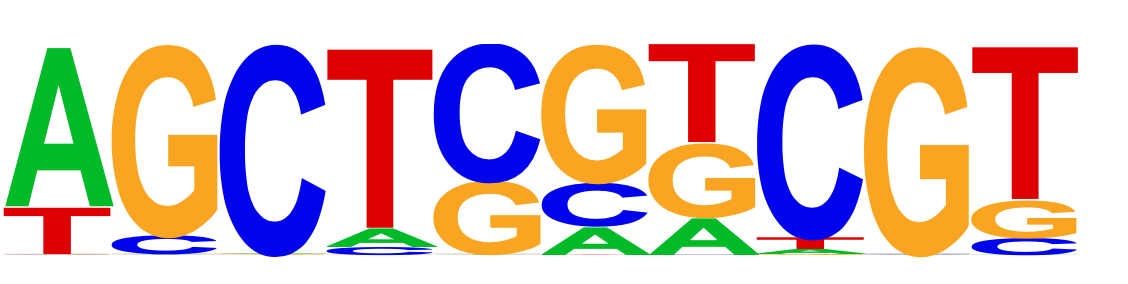 | 1e-10 | 1.67% | 0.45% |
| 31 * | 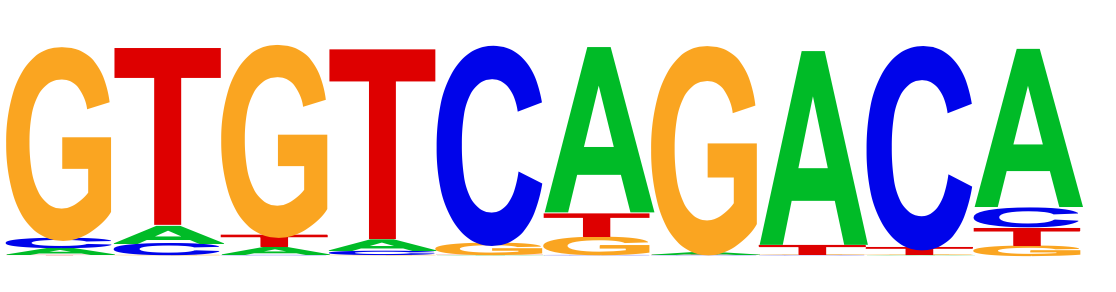 | 1e-10 | 1.71% | 0.47% |
| 32 * | 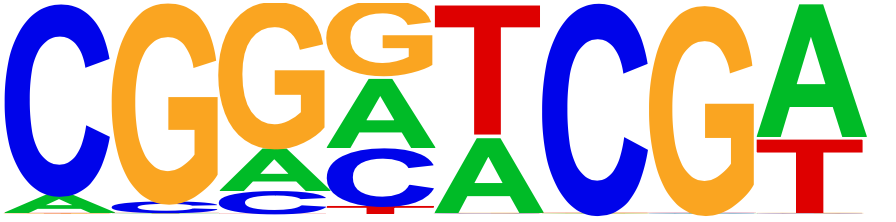 | 1e-10 | 5.93% | 3.20% |
| 33 * | 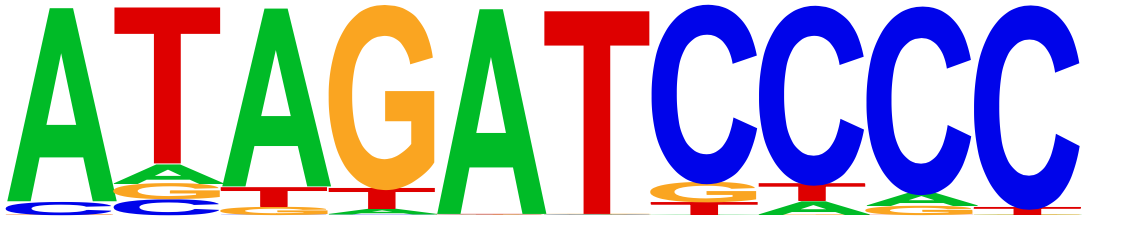 | 1e-10 | 1.27% | 0.27% |
| 34 * | 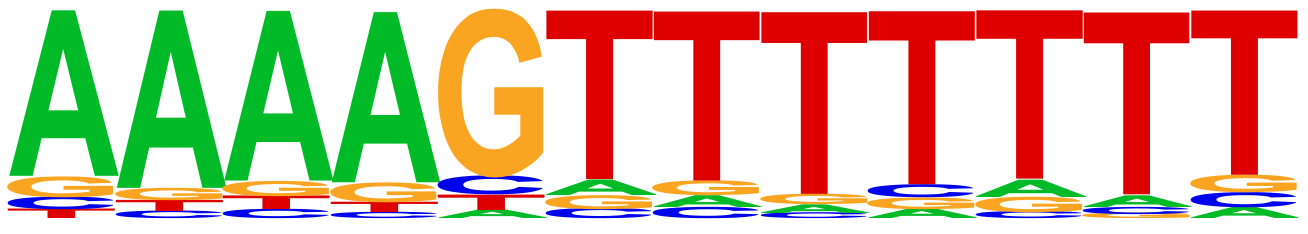 | 1e-10 | 2.63% | 0.99% |
| 35 * | 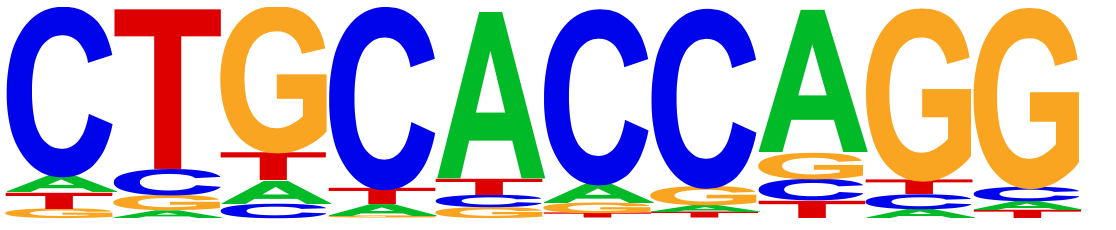 | 1e-10 | 3.91% | 1.81% |
| 36 * | 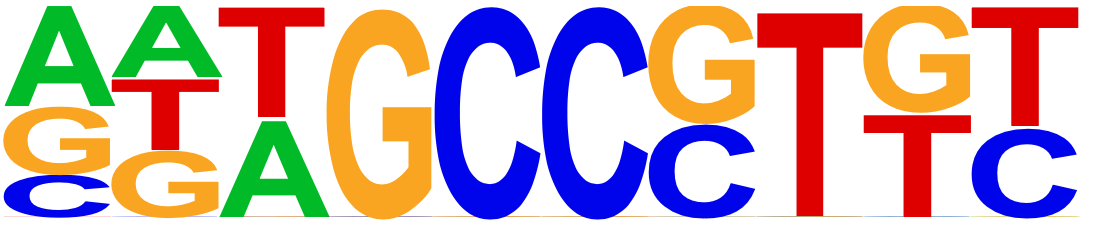 | 1e-10 | 4.61% | 2.29% |
| 37 * | 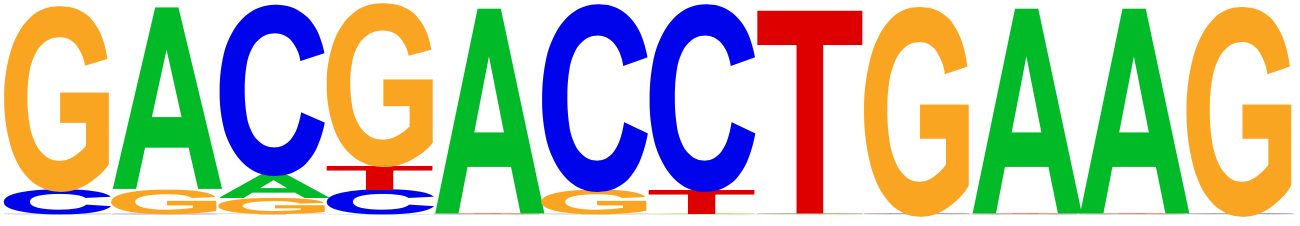 | 1e-9 | 0.35% | 0.01% |
| 38 * | 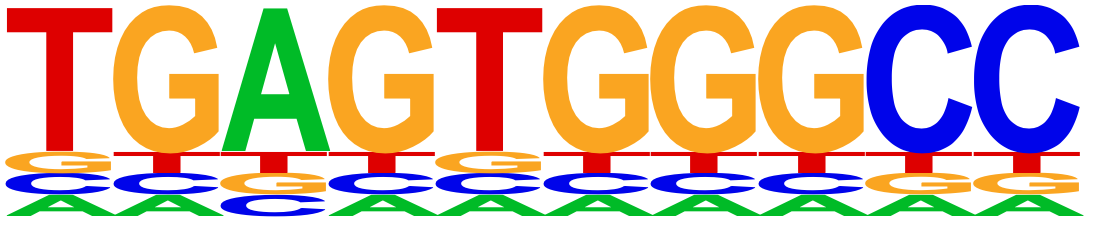 | 1e-9 | 98.16% | 95.79% |
| 39 * | 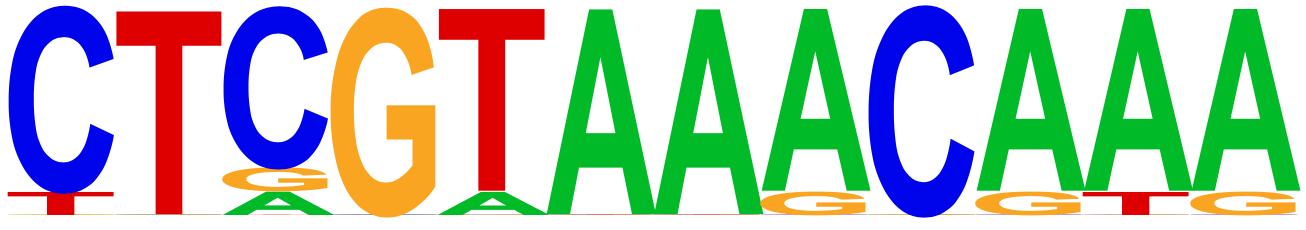 | 1e-9 | 0.40% | 0.02% |
| 40 * | 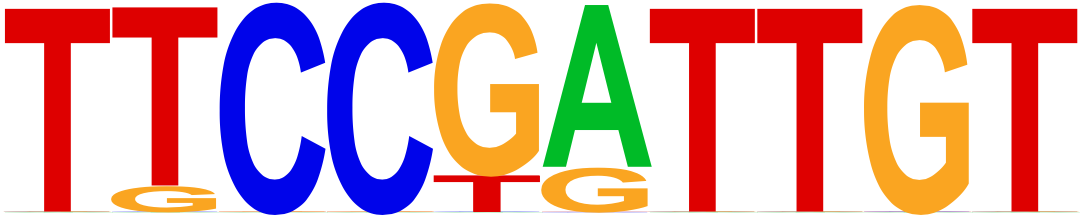 | 1e-9 | 0.75% | 0.10% |
| 41 * | 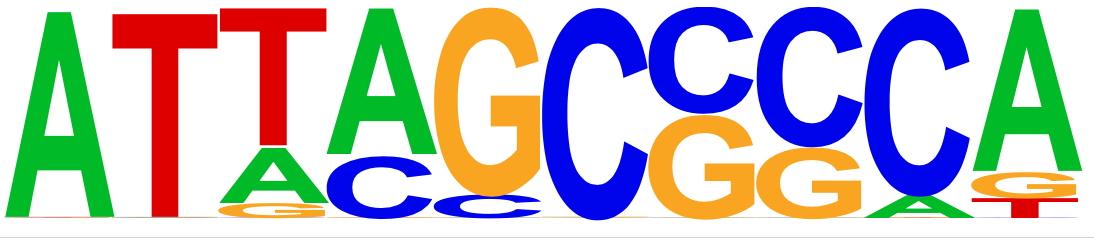 | 1e-7 | 0.70% | 0.11% |
| 42 * | 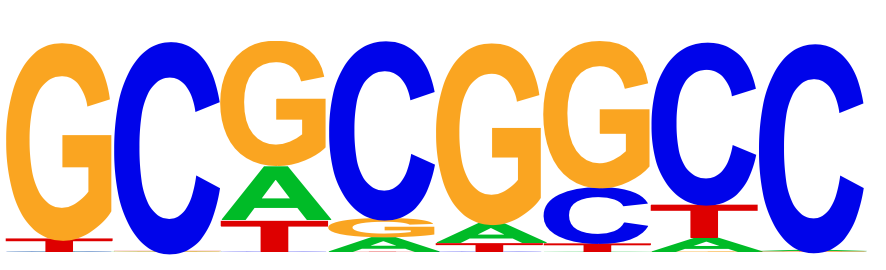 | 1e-7 | 7.73% | 5.13% |
| 43 * | 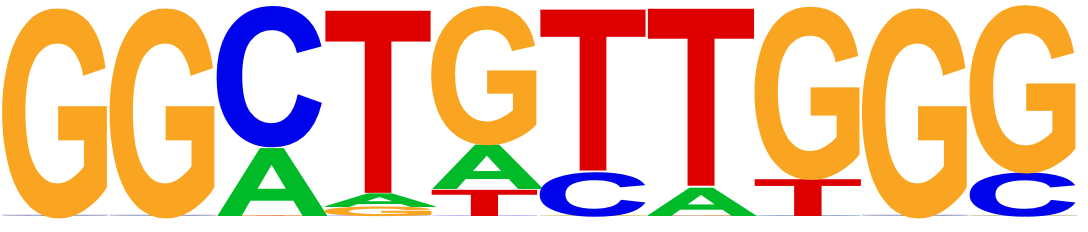 | 1e-6 | 1.01% | 0.27% |
| 44 * | 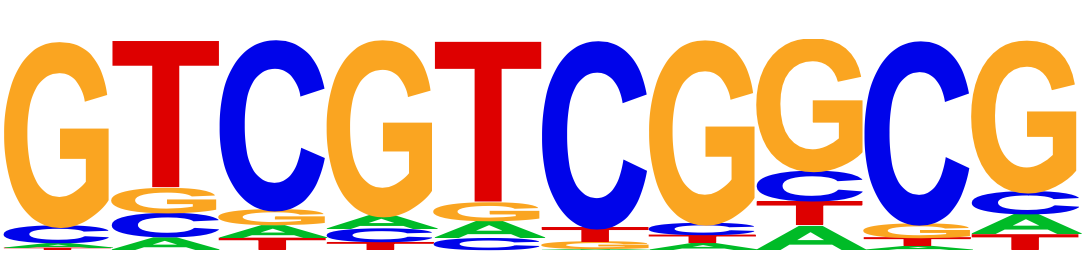 | 1e-6 | 7.73% | 5.19% |
| 45 * | 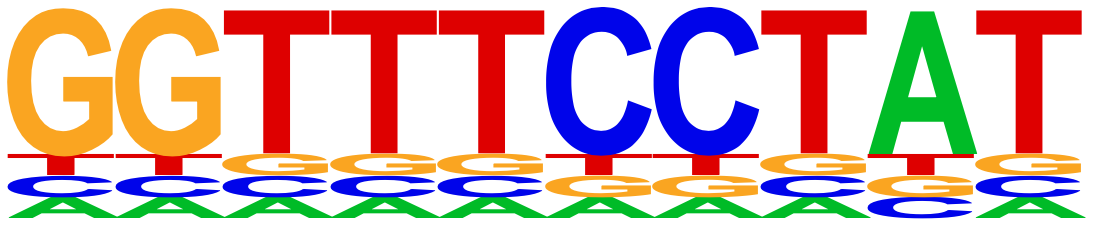 | 1e-6 | 0.26% | 0.01% |

**Note:** Total target sequences = 2278; Total background sequences = 47517; * - possible false positive.
